# Cytotoxic Acylphloroglucinol Derivatives from *Callistemon salignus*

**DOI:** 10.1007/s13659-017-0138-6

**Published:** 2017-06-15

**Authors:** Xu-Jie Qin, Tong Shu, Qian Yu, Huan Yan, Wei Ni, Lin-Kun An, Pan-Pan Li, Yin-E Zhi, Afsar Khan, Hai-Yang Liu

**Affiliations:** 1 State Key Laboratory of Phytochemistry and Plant Resources in West China, Kunming Institute of Botany, Chinese Academy of Sciences, Kunming, 650201 People’s Republic of China; 2 Yunnan Key Laboratory of Medicinal Chemistry, Kunming, 650201 People’s Republic of China; 3 University of Chinese Academy of Sciences, Beijing, 100049 People’s Republic of China; 4 Institute of Medicinal Chemistry and Chemical Biology, School of Pharmaceutical Sciences, Sun Yat-sen University, Guangzhou, 510006 People’s Republic of China; 5 Department of Chemistry, COMSATS Institute of Information Technology, Abbottabad, 22060 Pakistan

**Keywords:** *Callistemon salignus*, Myrtaceae, Meroterpenoids, Cytotoxicity

## Abstract

**Abstract:**

Callisalignenes G–I (**1**–**3**), three new meroterpenoids of *β*-triketone and monoterpene, along with two known analogues (**4** and **5**), were isolated from *Callistemon salignus*. Their structures and absolute configurations were unambiguously established by a combination of NMR and MS analysis and electronic circular dichroism (ECD) evidence. Callisalignenes H (**2**) and I (**3**) have a rare sec-butyl moiety at C-7. Meroterpenoids **1**–**3** exhibited cytotoxicity against HCT116 cells with IC_50_ values of 8.51 ± 1.8, 9.12 ± 0.3, and 16.33 ± 3.3 μM, respectively.

**Graphical Abstract:**

Cytotoxic Acylphloroglucinol Derivatives from *Callistemon salignus*

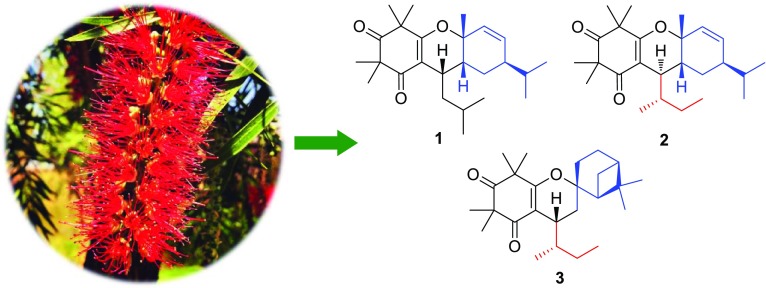

**Electronic supplementary material:**

The online version of this article (doi:10.1007/s13659-017-0138-6) contains supplementary material, which is available to authorized users.

## Introduction

**Fig. 1:**
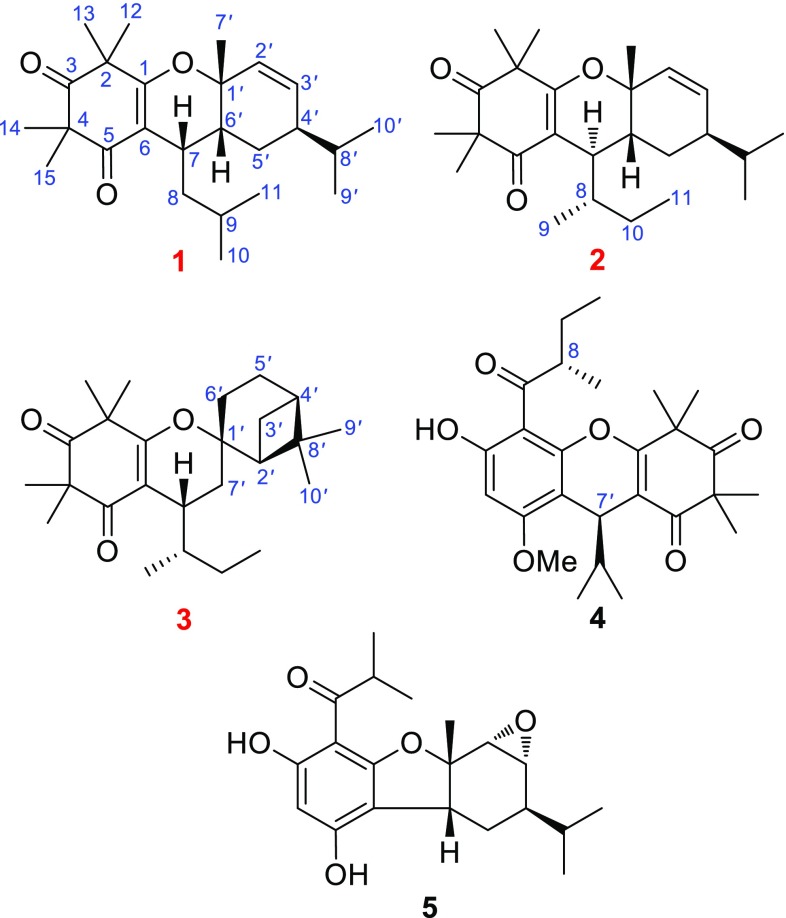
Structures of **1**–**5** obtained from *C. salignus*

Plants of genus *Callistemon* (Myrtaceae) are evergreen shrubs or small trees native to Australia and have been popularly cultivated in southern China as ornamental species with bottle brush inflorescence. Recently, acylphloroglucinols and their derivatives, including adducts of a phloroglucinol moiety coupled with its derivative or a terpenoid unit, have been extensively obtained from this genus [1–8]. Many of these compounds exhibited insecticidal [1], antibacterial [3], epoxide hydrolase inhibitory [4], and cytotoxic [5] effects. We have previously reported nine new acylphloroglucinol derivatives, callisalignones A–C and callisalignenes A–F, and 18 known analogues with antimicrobial and cytotoxic activities from the leaves and twigs of *Callistemon salignus* [9]. An extensive phytochemical investigation on petroleum ether extract of *C. salignus* resulted in the isolation of three acylphloroglucinol derivatives, named callisalignenes G–I (**1**–**3**), along with two known analogues (**4** and **5**) (Fig. 1). All the isolates were evaluated for their antimicrobial and cytotoxic activities. Herein, details of the isolation, structure elucidation, and bioactivity of these isolates are described.

## Results and Discussion

**Fig. 2:**
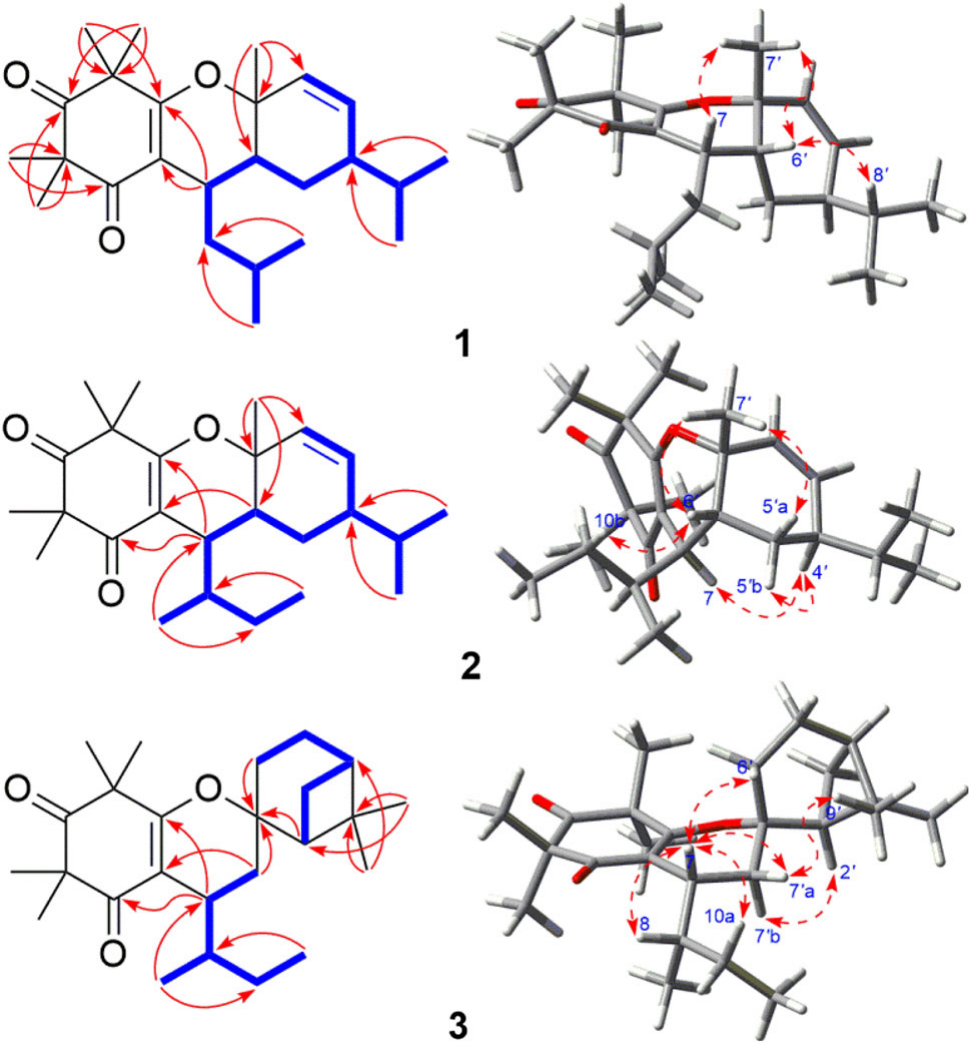
Key ^1^H–^1^H COSY, HMBC, and ROESY correlations of **1**–**3**

**Fig. 3:**
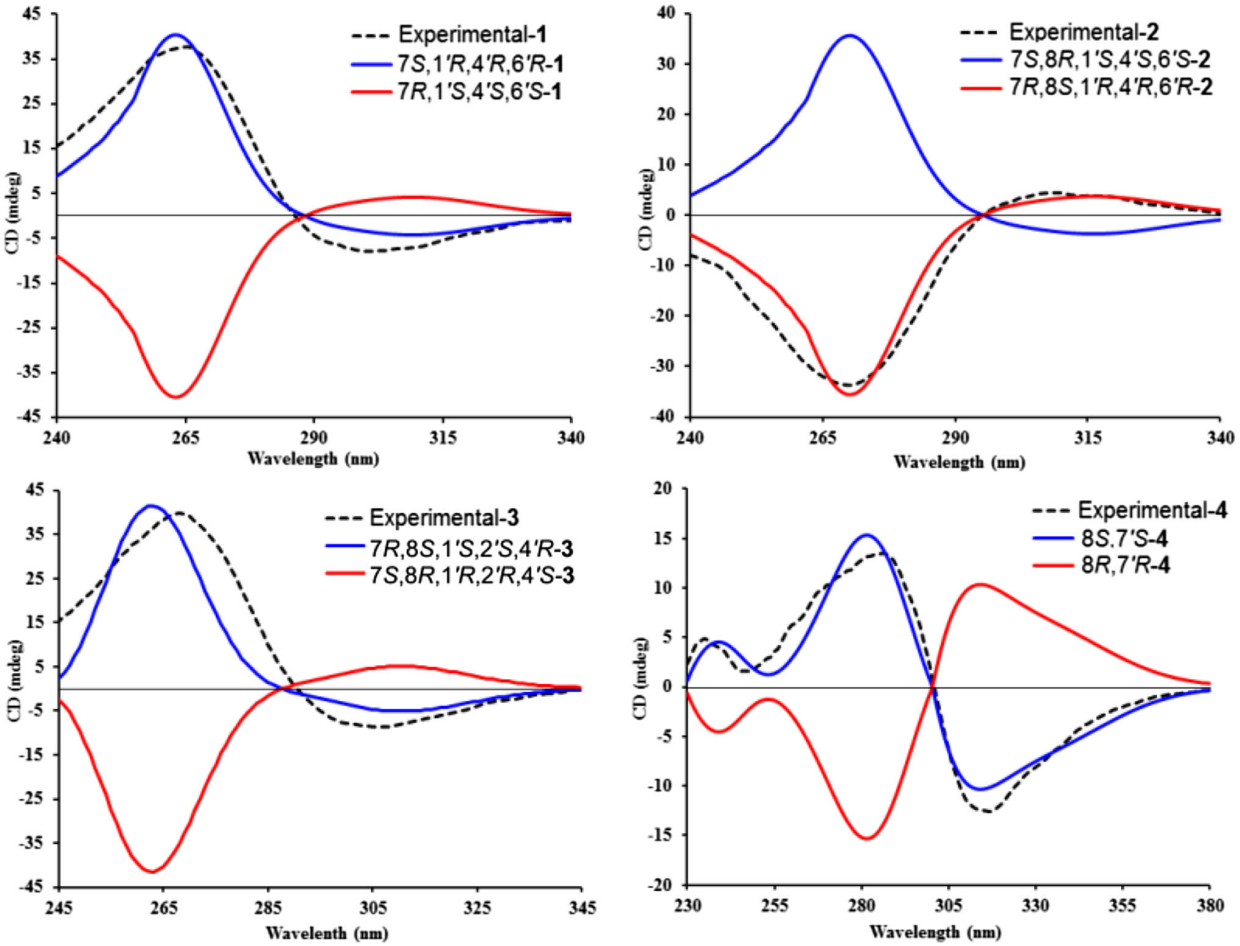
Calculated and experimental ECD spectra of **1**–**4**

Compound **1** was isolated as a colorless gum and its molecular formula was assigned as C_25_H_38_O_3_ by an ion peak at *m*/*z* 409.2714 [M + Na]^+^ (calcd for C_25_H_38_O_3_Na, 409.2713) in the HRESIMS and ^13^C NMR data. The UV absorption maximum at 265 nm combined with the IR absorption bands at 1721 and 1651 cm^−1^ revealed the presence of *α*,*β*-unsaturated keto group. The ^1^H NMR spectrum displayed signals for two olefinic protons at *δ*_H_ 5.75 (dd, *J* = 9.9, 1.8 Hz, H-2*′*) and 5.98 (dd, *J* = 9.9, 4.4 Hz, H-3*′*), five tertiary methyls at *δ*_H_ 1.26 (s, Me-7*′*), 1.28 (s, Me-12), 1.29 (s, Me-15), 1.31 (s, Me-14), and 1.35 (s, Me-13), and four secondary methyls at *δ*_H_ 0.94 (d, *J* = 6.9 Hz, Me-11), 0.95 (d, *J* = 6.9 Hz, Me-10), 0.98 (d, *J* = 6.8 Hz, Me-10*′*), and 0.99 (d, *J* = 6.8 Hz, Me-9*′*). The ^13^C NMR and HSQC spectra of **1** exhibited 25 carbon signals corresponding to nine methyls, two methylenes, seven methines (including two olefinic carbons at *δ*_C_ 130.9 and 135.2), and seven quaternary carbons (including two carbonyls at *δ*_C_ 198.6 and 213.4, two olefinic carbons at *δ*_C_ 110.3 and 167.0, and one oxygenated carbon at *δ*_C_ 76.2). The above-mentioned NMR data were similar to those of callistiviminene M [7], except for the presence of an additional methylene. The ^1^H–^1^H COSY spectrum disclosed the presence of a single spin system. The HMBC correlations from Me-12 and Me-13 to the carbonyl carbon C-3 (*δ*_C_ 213.4) and an oxygenated olefinic carbon C-1 (*δ*_C_ 167.0) and from Me-14 and Me-15 to the carbonyl carbons C-3 (*δ*_C_ 213.4) and C-5 (*δ*_C_ 198.6) revealed the presence of a *β*-triketone moiety. The isopentyl group was attached to the *β*-triketone, based on the HMBC correlations from H-7 (*δ*_H_ 2.97) to C-6 (*δ*_C_ 110.3) and the substructure of H-7–H_2_-8–H-9–Me-10/Me-11 suggested by ^1^H–^1^H COSY spectrum (Fig. 2). Similarly, the HMBC correlations from Me-7*′* to C-1*′* (*δ*_C_ 76.2), C-2*′* (*δ*_C_ 130.9), and C-6*′* (*δ*_C_ 33.7), as well as the substructure indicated by ^1^H–^1^H COSY spectrum and the molecular formula information, verified that the monoterpenoid moiety (*α*-phellandrene) and the *β*-triketone unit were connected via C-1–O–C-1*′* and C-7–C-6*′* bonds. The relative configuration of **1** was defined by a ROESY experiment. The ROESY correlations (Fig. 2) of Me-7*′* with H-6*′*, of H-8*′* with H-6*′*, and of H-7 with Me-7*′* suggested that these protons were cofacial. Finally, the absolute configuration of **1** was unambiguously determined as 7*S*,1*′R*,4*′R,*6*′R* from a positive Cotton effect at 265 (Δ*ε* +36.23) in its experimental ECD spectrum in comparison with the theoretical spectrum (Fig. 3). Therefore, structure of callisalignene G was established as **1**.

Compound **2** was assigned the same molecular formula as that of **1** by HRESIMS (*m*/*z* 409.2708 [M + Na]^+^, calcd for C_25_H_38_O_3_Na, 409.2713) and ^13^C NMR data. The ^1^H and ^13^C NMR spectra of **2** were similar to those of **1** except for the signals of a side chain at C-7. The observed HMBC correlations from Me-11 (*δ*_H_ 0.82) to C-8 (*δ*_C_ 39.2), and from Me-9 (*δ*_H_ 0.95) to C-7 (*δ*_C_ 36.4) and C-10 (*δ*_C_ 24.7) supported that a sec-butyl group was attached to C-7 in **2** rather than that of isobutyl in **1**. After the full assignment of ^1^H and ^13^C NMR data for **2** by HMBC and ^1^H–^1^H COSY spectra (Fig. 2), the gross structure of the other parts was the same as **1**. However, the carbon signals for C-1 (*δ*_C_ 79.4), C-4*′* (*δ*_C_ 36.7), and C-6 (*δ*_C_ 39.8) of **2** were very different, implying that **2** was an isomer of **1**. The ROESY correlations of Me-7*′* with H-6*′* and H-5*′*a indicated that these protons were in the same plane, while those of H-5*′*b with H-4*′*, and of H-4*′* with H-7 suggested that they were co-facial. The assignment of 8*S* configuration could be explained in view of its proposed biosynthetic pathway [9]. Furthermore, the absolute configuration of **2** was determined as 7*R*,8*S*,1*′R*,4*′R*,6*′R* by comparing the calculated ECD spectrum with its experimental values (Fig. 3). Thus, the structure of callisalignene H was determined as **2**.

Compound **3** had the same molecular formula C_25_H_38_O_3_ as those of **1** and **2** by HRESIMS (387.2899 [M + H]^+^, calcd for C_25_H_39_O_3_, 387.2894) and ^13^C NMR data. Comparison of the NMR data of **3** (Table 1) with those of **2** suggested that they both shared the same *β*-triketone moiety with an isobutyl group at C-7. The remaining ten carbon signals for the monoterpene unit corresponded to two tertiary methyls, four methylenes, two methines, and two quaternary carbons (including an oxygenated one) revealed the presence of *β*-pinene unit. This conclusion was further confirmed by HMBC correlation from Me-9*′* (*δ*_H_ 0.99) and Me-10*′* (*δ*_H_ 1.31) to C-2*′* (*δ*_C_ 52.9), C-4*′* (*δ*_C_ 40.4) and C-8*′* (*δ*_C_ 32.6), from H-2*′* (*δ*_H_ 2.04), H-6*′* (*δ*_H_ 1.88) and H-7*′* (*δ*_H_ 1.89) to C-1*′*, and the fragment (H-2*′*–H_2_-3*′*–H-4*′*–H_2_-5*′*–H_2_-6*′*) determined by ^1^H–^1^H COSY spectrum (Fig. 2). In the ROESY spectrum of **3**, correlations of H-2*′α* with H-7*′*b and of H-7*′*a with H-7 indicated that H-7 was *β*-oriented. From biosynthetic considerations, the absolute configuration of C-8 in **3** was also assigned as *S*. The absolute configuration of **3** was assigned as 7*R*,8*S*,1*′S*,2*′S*,4*′R* by comparison of its experimental and calculated ECD spectra (Fig. 3). Accordingly, the structure of callisalignene I was established as **3**.

**Table 1:** ^1^H (800 MHz) and ^13^C (200 MHz) NMR data for **1**–**3** in CDCl_3_

No.	**1**		**2**		**3**	
	*δ*_H_ (mult., *J* in Hz)	*δ*_C_	*δ*_H_ (mult., *J* in Hz)	*δ*_C_	*δ*_H_ (mult., *J* in Hz)	*δ*_C_
1		167.0, C		170.8, C		170.2, C
2		47.9, C		47.5, C		48.3, C
3		213.4, C		213.7, C		213.8, C
4		55.9, C		55.7, C		55.4, C
5		198.6, C		197.8, C		197.8, C
6		110.3, C		116.1, C		112.4, C
7	2.97, ddd (11.2, 6.2, 3.8)	28.2, CH	2.68, dd (5.2, 3.4)	36.4, CH	2.86, ddd (11.8, 6.5, 3.8)	30.7, CH
8	a 1.97, br t (3.0)	35.2, CH_2_	1.60, overlapped	39.2, CH	2.44, m	32.6, CH
	b 1.10, ddd (13.9, 11.7, 2.6)					
9	1.61, m	24.7, CH	0.95, d (6.9)	17.6, CH_3_	0.58, d (6.9)	13.4, CH_3_
10	0.95, d (6.9)	21.5, CH_3_	a 1.24, m	24.7, CH_2_	a 1.31, overlappled	27.8, CH_2_
			b 1.03, m		b 1.23 m	
11	0.94, d (6.9)	24.2, CH_3_	0.82, t (8.2)	12.6, CH_3_	0.95, t (7.4)	12.3, CH_3_
12	1.28, s	25.8, CH_3_	1.32, s	24.6, CH_3_	1.33 s	25.4, CH_3_
13	1.35, s	23.5, CH_3_	1.39, s	25.1, CH_3_	1.38 s	24.5, CH_3_
14	1.31, s	21.6, CH_3_	1.29, s	23.8, CH_3_	1.34, s	23.4, CH_3_
15	1.29, s	26.8, CH_3_	1.32, s	25.1, CH_3_	1.32, s	25.9, CH_3_
1*′*		76.2, C		79.4, C		84.2, C
2*′*	5.75, dd (9.9, 1.8)	130.9, CH	5.26, dd (10.2, 2.3)	130.6, CH	2.04, t (5.5)	52.9, CH
3*′*	5.98 dd (9.9, 4.4)	135.2, CH	5.69, dd (10.2, 2.3)	134.5, CH	a 1.86, m	26.9, CH_2_
					b 1.66, t (11.1)	
4*′*	1.95, m	41.2, CH	2.15, m	36.7, CH	1.98, m	40.4, CH
5*′*	a 1.67, br d (13.8)	20.8, CH_2_	a 1.67, br t (4.8)	30.1, CH_2_	a 2.30, dt (10.1, 6.0)	27.0, CH_2_
	b 1.29, br t (7.1)		b 1.63, dd (10.1, 3.8)		b 1.63, br d (10.3)	
6*′*	1.86, ddd (13.2, 6.3, 2.8)	33.7, CH	2.19, q (4.1)	39.8, CH	1.88, 2H m	25.0, CH_2_
7*′*	1.26, s	23.8, CH_3_	1.50, s	27.9, CH_3_	a 1.89, dd (13.4, 6.5)	33.4, CH_2_
					b 1.34, overlapped	
8*′*	1.63, m	31.5, CH	1.59, m	31.6, CH		38.2, C
9*′*	0.99, d (6.8)	21.0, CH_3_	0.88, d (6.6)	19.4, CH_3_	0.99, s	23.4, CH_3_
10*′*	0.98, d (6.8)	20.8, CH_3_	0.87, d (6.6)	19.3, CH_3_	1.31, s	27.5, CH_3_

Besides the three new meroterpenoids, two known compounds were identified as (–)-callistenone F (**4**) [10] and viminalin B (**5**) [5] by the comparison of their experimental data and reported values. It is to be noted that callistenone K has been reported as a racemic mixture with a specific rotation value of +3.6 (*c* 1.0, CHCl_3_), but in the current study **4** was isolated as a single diastereomer with a negative specific rotation value [−133.0 (*c* 0.1, MeOH)]. Further analysis of its experimental and calculated ECD spectra (Fig. 3) validated the absolute configurations of 8*S*,7*′S* for (–)-**4**.

All the isolates were evaluated for their antimicrobial effects toward three bacterial (*S. aureus*, *E. coli*, and *P. aeruginosa*) and three fungal strains (*A. fumigatus*, *C. parapsilosis*, and *C. albicans*). None of them showed antimicrobial effects (MIC >250 μg/mL). Additionally, cytotoxicities of **1**–**5** against six human cancer cells (HCT116, Huh7, Hela, CCRF-CEM, DU145, and A549) were also conducted and the results were summarized in Table 2. **1**–**3** exhibited cytotoxicity against HCT116 cells with IC_50_ values of 8.51 ± 1.8, 9.12 ± 0.3, and 16.33 ± 3.3 μM, respectively, compared to that of positive control (VP-16, 20.26 ± 0.5 μM). Moreover, **1** and **3** displayed cytotoxicity against A549 cells with IC_50_ values of 12.85 ± 8.2 and 10.03 ± 3.2 μM (VP-16, 25.79 ± 6.2 μM), respectively.

## Experimental

### General Experimental Procedures

Optical rotations were measured on a Jasco P-1020 polarimeter. UV spectra were recorded on a Shimadzu UV2401 PC spectrophotometer. IR spectra were determined on Bruker FT-IR Tensor-27 infrared spectrophotometer with KBr discs. ECD spectra were recorded on an Applied Photophysics spectropolarimeter. 1D and 2D NMR spectra were recorded on Bruker AV 600 or 800 MHz spectrometers using TMS as an internal standard. Chemical shifts (*δ*) were expressed in ppm with reference to the solvent signals, and coupling constant (*J*) values were reported in Hz. HRESIMS data were measured using an Agilent 1290 UPLC/6540 Q-TOF mass spectrometer. Sephadex LH-20 (GE Healthcare, Uppsala, Sweden), Si gel (200–300 mesh, Qingdao Marine Chemical Co., Qingdao, People’s Republic of China), and RP-18 (50 μm, Merck, Germany) were used for column chromatography (CC). Semi-preparative HPLC was performed on an Agilent 1260 instrument with a ZORBAX SB-C18 column (9.4 × 250 mm, 5 μm). Fractions were monitored by Si gel GF_254_ (Qingdao Marine Chemical Co., China) or RP-18 F_254_ (Merck, Darmstadt, Germany) plates. Spots were visualized under UV light and by spraying with 10% H_2_SO_4_ in EtOH followed by heating.

### Plant Material

Twigs and leaves of *C. salignus* were collected from Kunming City, Yunnan Province, P. R. China, in February 2016 and identified by Dr. Rong Li (Kunming Institute of Botany, Chinese Academy of Sciences). A voucher specimen (HY0025) was deposited in the State Key Laboratory of Phytochemistry and Plant Resources in West China, Kunming Institute of Botany, Chinese Academy of Sciences.

### Extraction and Isolation

Air-dried and powdered twigs and leaves of *C. salignus* (10.0 kg) were percolated with petroleum ether (PE) at room temperature three times (3 × 24 h; 50 L) and then filtered. After removal of solvent under reduced pressure, the crude extract (130 g) was subjected to silica gel CC, eluted with PE-EtOAc (100:1 → 1:1, v/v) to yield five fractions A–E. Fraction A (25 g) was applied to Sephadex LH-20 column (CHCl_3_–MeOH 1.5:1, v/v) to give A3 (3 g), which was further separated on an RP-18 column and eluted with a gradient of MeCN–H_2_O (80:20 → 95:5, v/v) to obtain five subfractions (A_3-1_–A_3-5_). Subfraction A_3-4_ (225 mg) was subsequently purified by semi-preparative HPLC with MeCN–H_2_O (90:10 → 100:0 v/v, 5 mL/min) as mobile phase to afford **1** (5 mg), **2** (4 mg), **3** (8 mg), and **5** (35 mg). Similarly, fraction C (42 g) was separated by Sephadex LH-20 column (CHCl_3_–MeOH 1.5:1, v/v) to give three subfraction (C_1_–C_3_). After repeated purification by RP-18 column with MeCN–H_2_O (60:40 → 75:25, v/v), **4** (18 mg) was obtained from C_2_ (0.5 g).

#### Callisalignene G (**1**)

Colorless gum; [*α*] +188.3 (*c* 0.1, MeOH); UV (MeOH) *λ*_max_ (log *ε*) 265 (4.18) nm; CD (MeOH) 265 (Δ* ε* + 36.23), 306 (Δ*ε* −7.57) nm; IR (KBr) *v*_max_ 3440, 2932, 1721, 1651, 1469, 1248 cm–^1^; ^1^H and ^13^C NMR data, see Table 1; HRESIMS *m*/*z* 409.2714 [M + Na]^+^ (calcd for C_25_H_38_O_3_Na, 409.2713).

#### Callisalignene H (**2**)

Colorless gum; [*α*] −221.4 (*c* 0.1, MeOH); UV (MeOH) *λ*_max_ (log *ε*) 266 (4.20) nm; CD (MeOH) 267 (Δ*ε* −31.35), 309 (Δ*ε* + 4.11) nm; IR (KBr) v_max_ 3439, 2930, 1720, 1649, 1467, 1247 cm^−1^; ^1^H and ^13^C NMR data, see Table 1; HRESIMS *m*/*z* 409.2708 [M + Na]^+^ (calcd for C_25_H_38_O_3_Na, 409.2713).

#### Callisalignene I (**3**)

Colorless gum; [*α*] +164.7 (*c*, MeOH); UV (MeOH) *λ*_max_ (log *ε*) 267 (4.24) nm; CD (MeOH) 267 (Δ*ε* +27.50), 306 (Δ*ε* −6.02) nm; IR (KBr) *v*_max_ 3428, 2962, 1718, 1651, 1467, 1384, 1174^−1^; ^1^H and ^13^C NMR data, see Table 1; HRESIMS *m*/*z* 387.2899 [M + H]^+^ (calcd for C_25_H_39_O_3_, 387.2894).

#### (–)-Callistenone K (**4**)

Colorless gum; [*α*] −133.0 (*c* 0.1, MeOH); CD (MeOH) 235 (Δ*ε* +5.45), 248 (Δ*ε* +1.77), 286 (Δ*ε* +15.10), 317 (Δε −13.95) nm.

### Quantum Chemical ECD Calculations

The conformations generated by the MM2 force field in Chem-Bio3D software overlaid with key correlations observed in the ROESY spectrum were subjected to semi-empirical PM3 quantum mechanical geometry optimizations using the Gaussian 09 program [11]. The corresponding minimum geometries were further optimized by Density Functional Theory (DFT) calculations at the B3LYP/6-31+G(d) level Table 2. The theoretical calculations of ECD were performed using Time Dependent DFT at B3LYP/6-311++G(2d,p) level in MeOH. The calculated ECD curves were generated by SpecDis (version 1.63) software [12].

**Table 2:** Cytotoxicities with IC_50_ values (μM) of meroterpenoids **1**–**5**

	HCT116	Huh7	Hela	CCRF-CEM	DU145	A549
**1**	8.51 ± 1.8	44.41 ± 3.2	36.46 ± 8.4	4.52 ± 1.3	39.92 ± 6.8	12.85 ± 8.2
**2**	9.12 ± 0.3	42.11 ± 3.6	46.99 ± 8.7	6.20 ± 0.8	18.44 ± 9.5	26.61 ± 6.4
**3**	16.33 ± 3.3	56.13 ± 7.3	62.27 ± 5.1	30.66 ± 4.6	36.24 ± 7.0	10.03 ± 8.2
**4**	31.14 ± 8.68	>100	>100	68.51 ± 8.7	22.14 ± 0.4	49.92 ± 5.5
**5**	93.35 ± 2.5	68.62 ± 7.8	66.18 ± 14.3	67.10 ± 0.9	>100	56.98 ± 4.6
VP-16	20.26 ± 0.5	7.43 ± 1.3	11.57 ± 3.2	1.11 ± 0.4	5.22 ± 1.9	25.79 ± 6.2

### Antibacterial Assay

The antimicrobial activities compounds **1**–**5** were carried out against three bacterial strains (*Staphylococcus aureus*, *Escherichia coli*, and *Pseudomonas aeruginosa*) and three fungal strains (*Aspergillus fumigatus, Candida parapsilosis, and C. albicans*) using the antimicrobial susceptibility assay [13]. The compounds were dissolved in DMSO, serially diluted to the concentration ranges of 250–0.061 μg/mL, and tested in a 96-well plate. Gentamycin (for bacteria) and voriconazole (for fungi) were used as positive controls. The experiments were conducted for three independent replicates. The MIC was determined as the lowest concentration that inhibited the visible growth of bacteria and fungi.

### Cytotoxicity Assay

All the compounds were evaluated for their cytotoxicities against six tumor cell lines, including HCT116 (human colorectal cancer cell line), Huh7 (human hepatoma cell line), Hela (human cervical cancer cell line), CCRF-CEM (human acute lymphocytic leukemia cell line), DU145 (human prostatic cancer cell line), and A549 (human lung cancer cell line), by MTT assay in 96-well plates [14]. VP-16 (etoposide) was used as a positive control.

## Electronic supplementary material

Below is the link to the electronic supplementary material.

Supplementary material 1 (DOCX 12178 kb)
