# Supplementary material for: Cytotoxic Acylphloroglucinol Derivatives from Callistemon salignus
Source: Nat Prod Bioprospect. 2017 Jun 15;7(4):315–21. doi: 10.1007/s13659-017-0138-6 (PMC5507812; doi:10.1007/s13659-017-0138-6)
Supplement: Supplementary file 1 — Supplementary material 1 (DOCX 12178 kb) [file 13659_2017_138_MOESM1_ESM.docx]

**Supplementary Information**

**Cytotoxic acylphloroglucinol derivatives from *Callistemon salignus***

Xu-Jie Qin ^a,b,1^, Tong Shu ^a,b,c,1^, Qian Yu ^d,1^, Huan Yan ^a,b^, Wei Ni ^a,b^, Lin-Kun An ^d^, Pan-Pan Li ^a,b^, Yin-E Zhi ^a, b^, Afsar Khan ^e^, Haiyang Liu ^a,b,^*

^a^ *State Key Laboratory of Phytochemistry and Plant Resources in West China, Kunming Institute of Botany, Chinese Academy of Sciences,* *Kunming 650201, People’s Republic of China*

^b^ *Yunnan Key Laboratory of Medicinal Chemistry, Kunming 650201, People’s Republic of China*

^c^ *University of Chinese Academy of Sciences, Beijing 100049, People’s Republic of China*

^d^ *Institute of Medicinal Chemistry and Chemical Biology, School of Pharmaceutical Sciences, Sun*

*Yat-sen University, Guangzhou 510006,* *People’s Republic of China*

^e^ *Department of Chemistry, COMSATS Institute of Information Technology, Abbottabad-22060,*

*Pakistan*

* Corresponding author.

*E-mail address*: haiyangliu@mail.kib.ac.cn (H. Y. Liu).

^1^ These authors contributed equally to this work.

**Contents** **of Supplementary material**  **Page**

Figures S1–S7. NMR and HRMS spectra of compound **1**…………………………………3‒6

Figures S8–S14. NMR and HRMS spectra of compound **2**………………………………...6‒9

Figures S15–S21. NMR and HRMS spectra of compound **3**…………………….………….10‒13

Computational data of **1**……………………………………………………………..…………14‒15

Computational data of **2**……………………………………………………………..…………16‒17

Computational data of **3**……………………………………………………………..…………18‒19

Computational data of **4**……………………………………………………………..…………20‒21


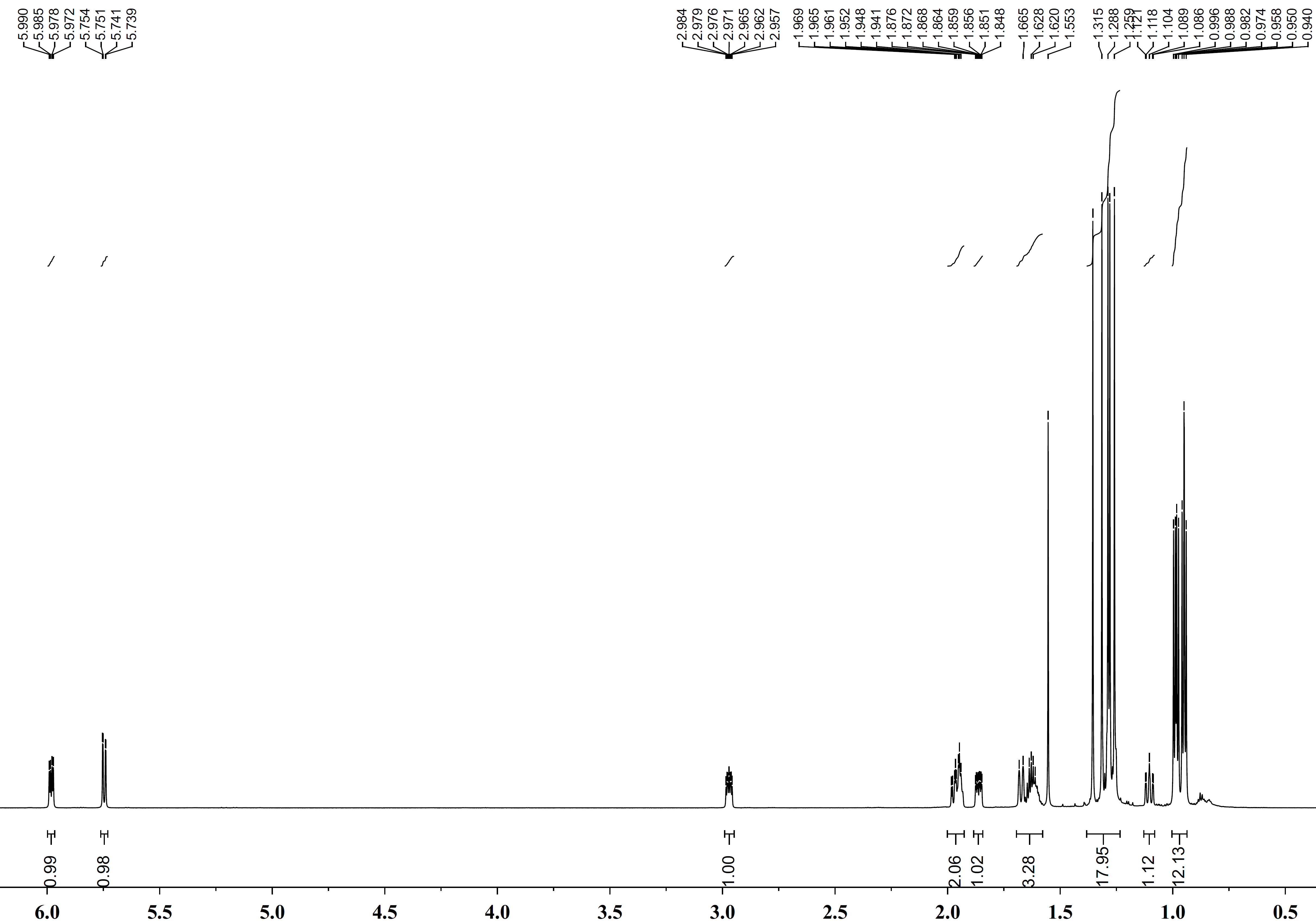


Figure S1. ^1^H NMR spectrum of compound **1** in CDCl_3_


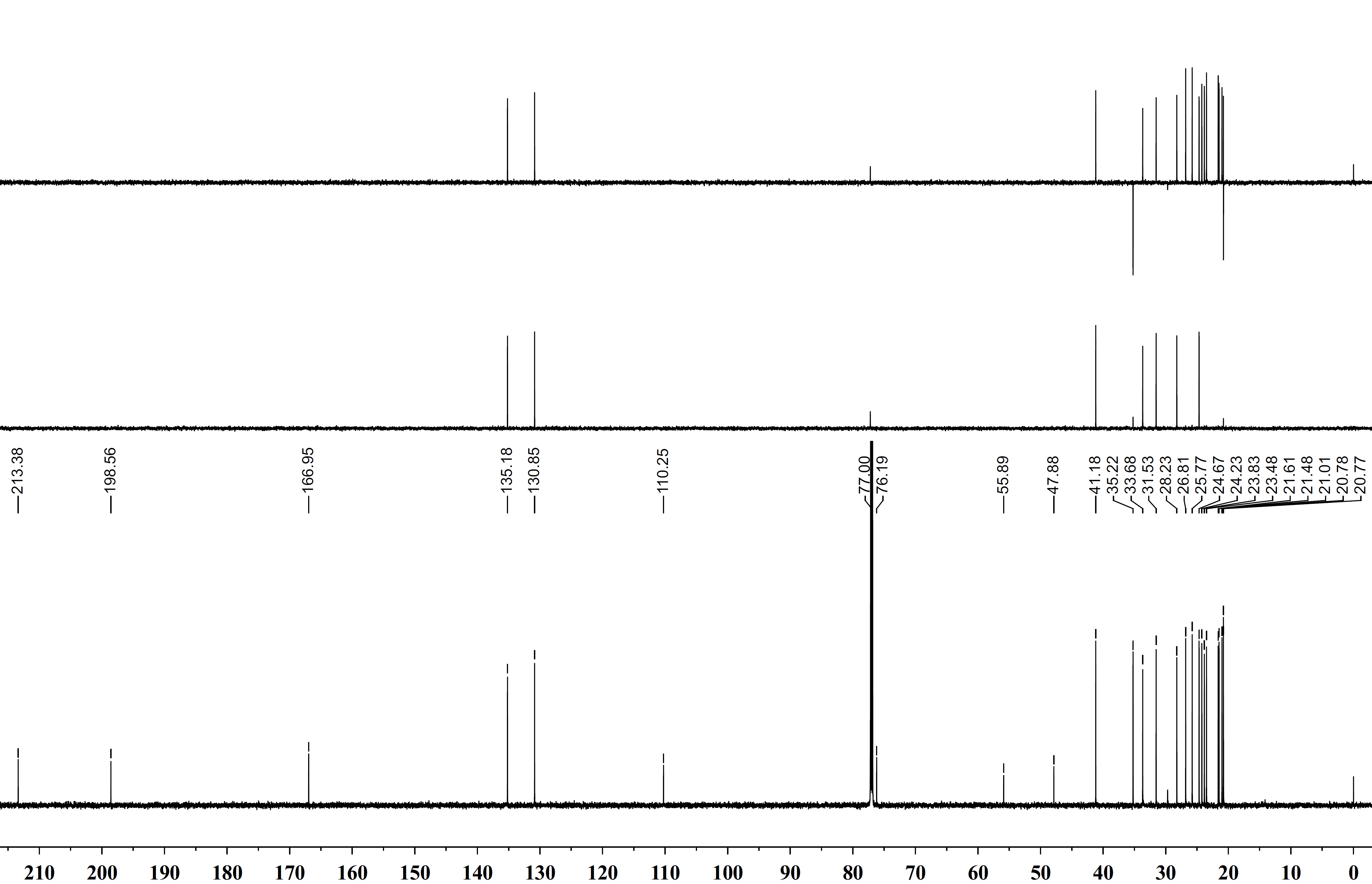


Figure S2. ^13^C NMR spectrum of compound **1** in CDCl_3_


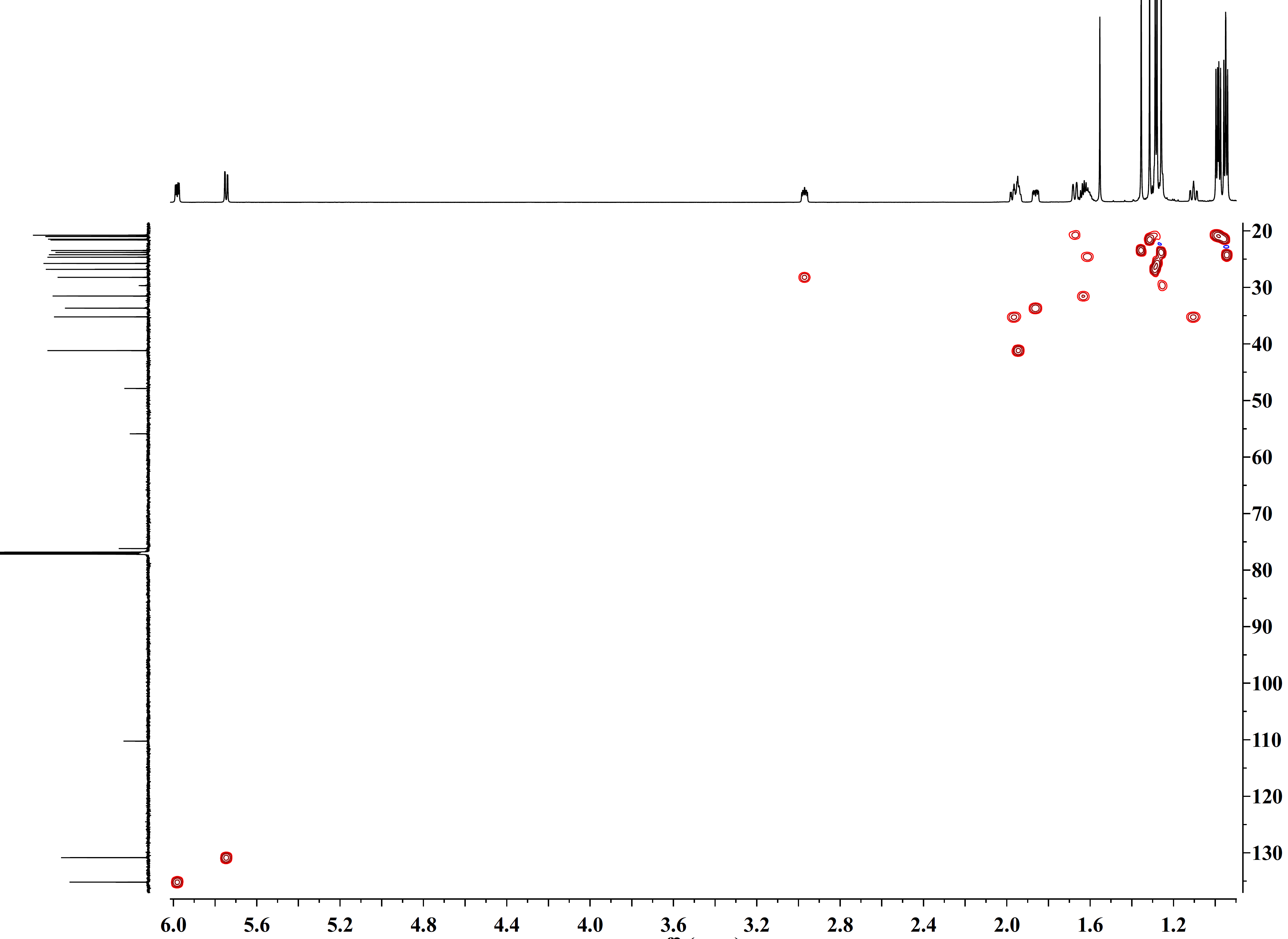


Figure S3. HSQC spectrum of compound **1**


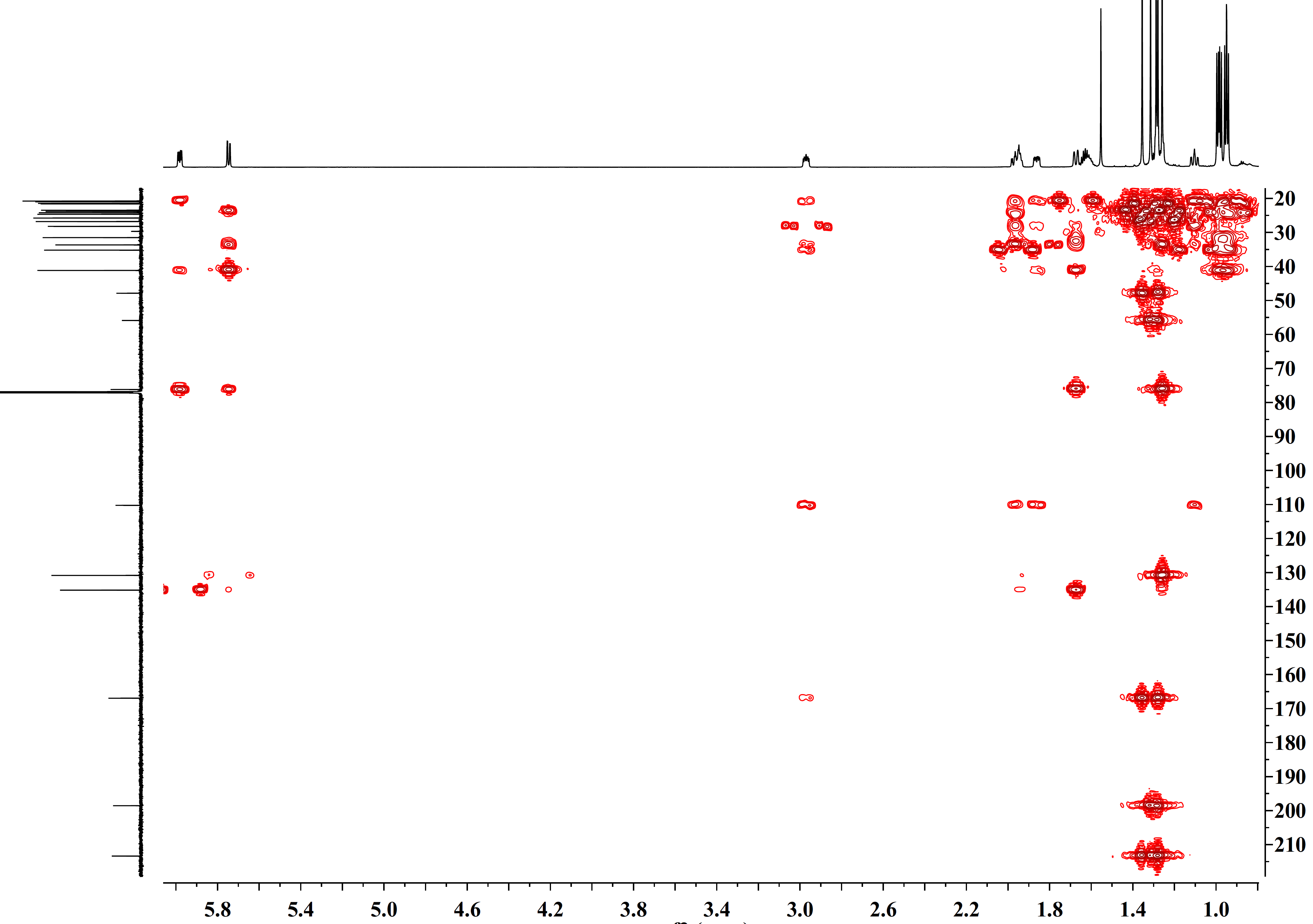


Figure S4. HMBC spectrum of compound **1**


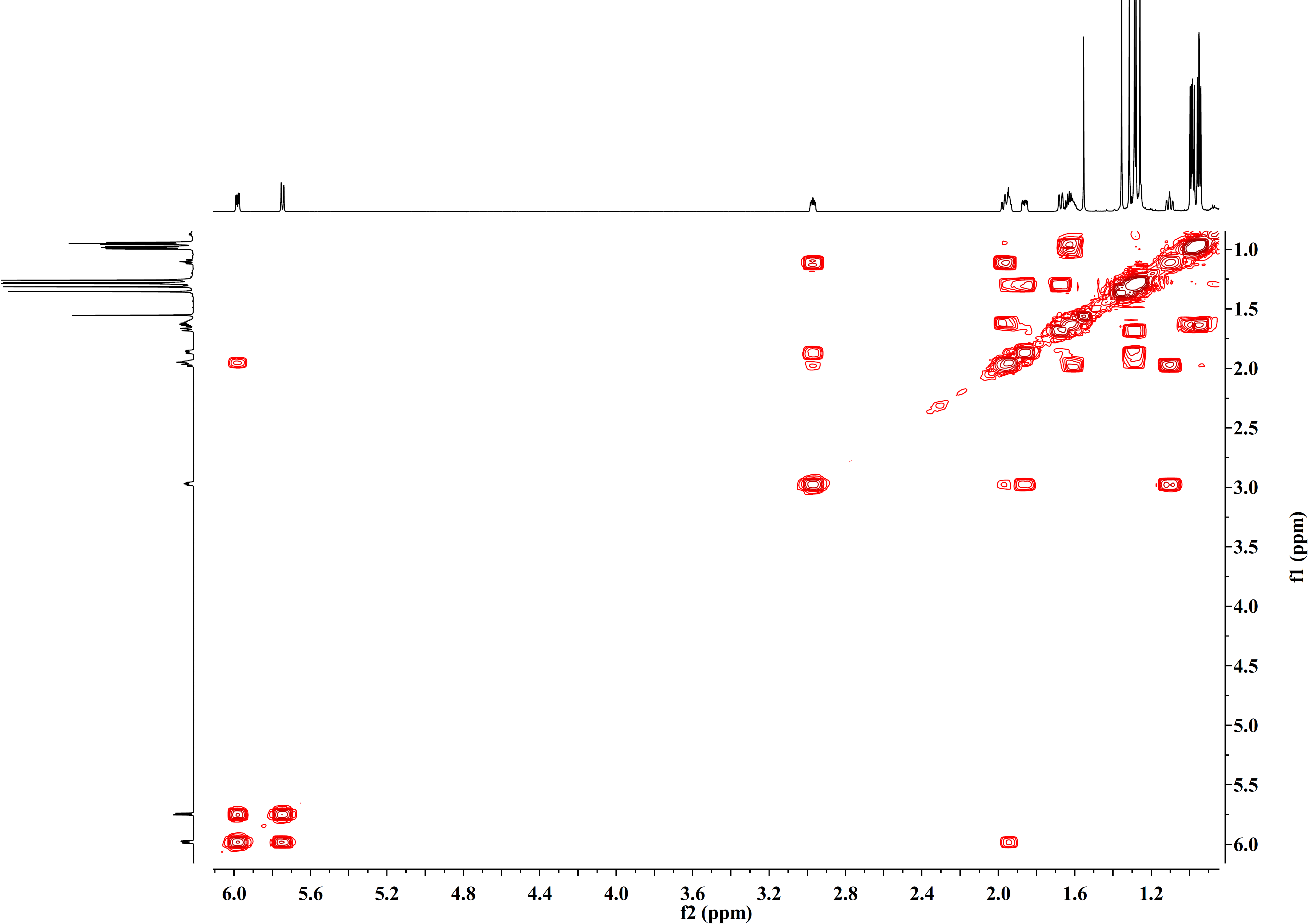


Figure S5. ^1^H‒^1^H COSY spectrum of compound **1**


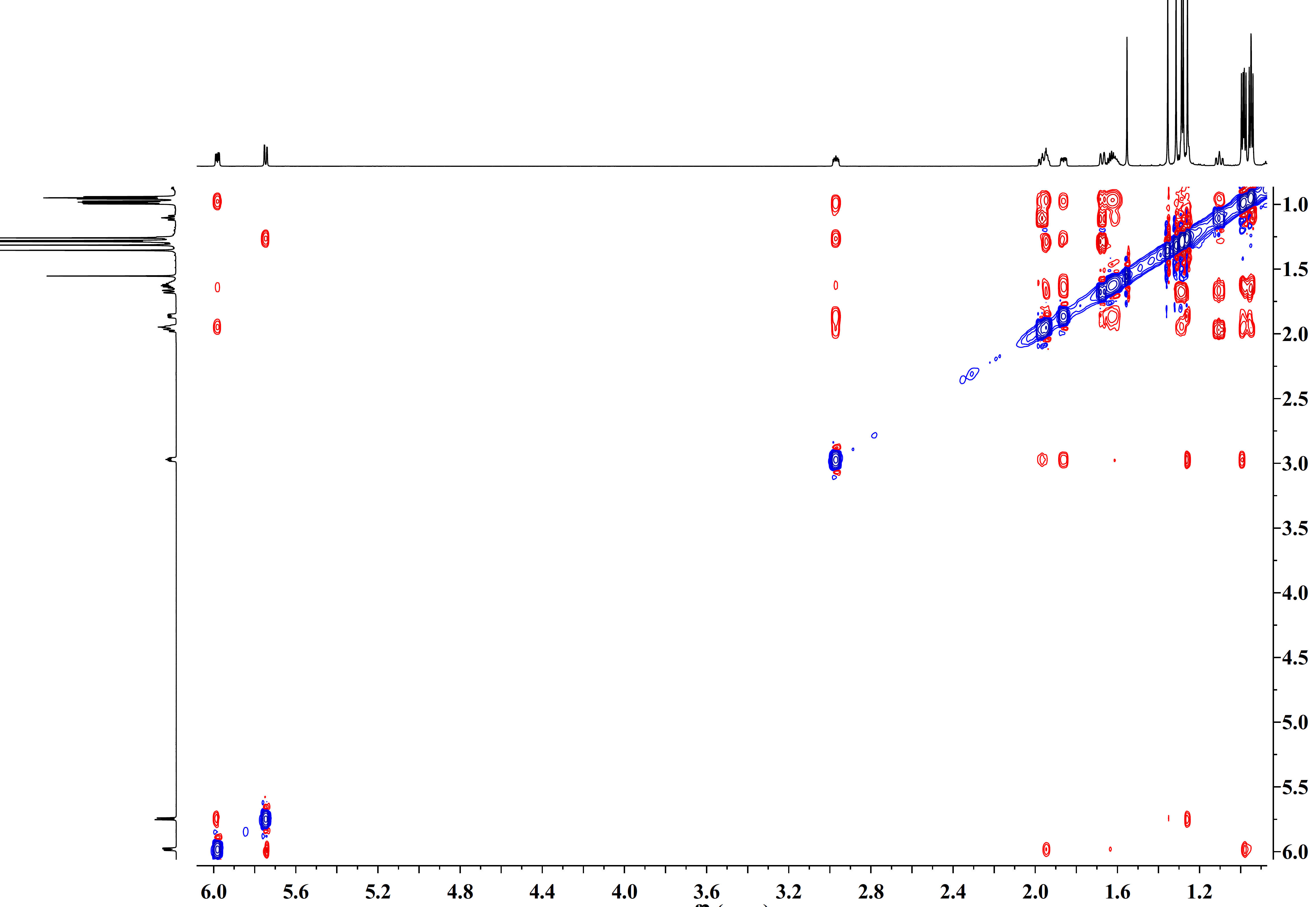


Figure S6. ROESY spectrum of compound **1**


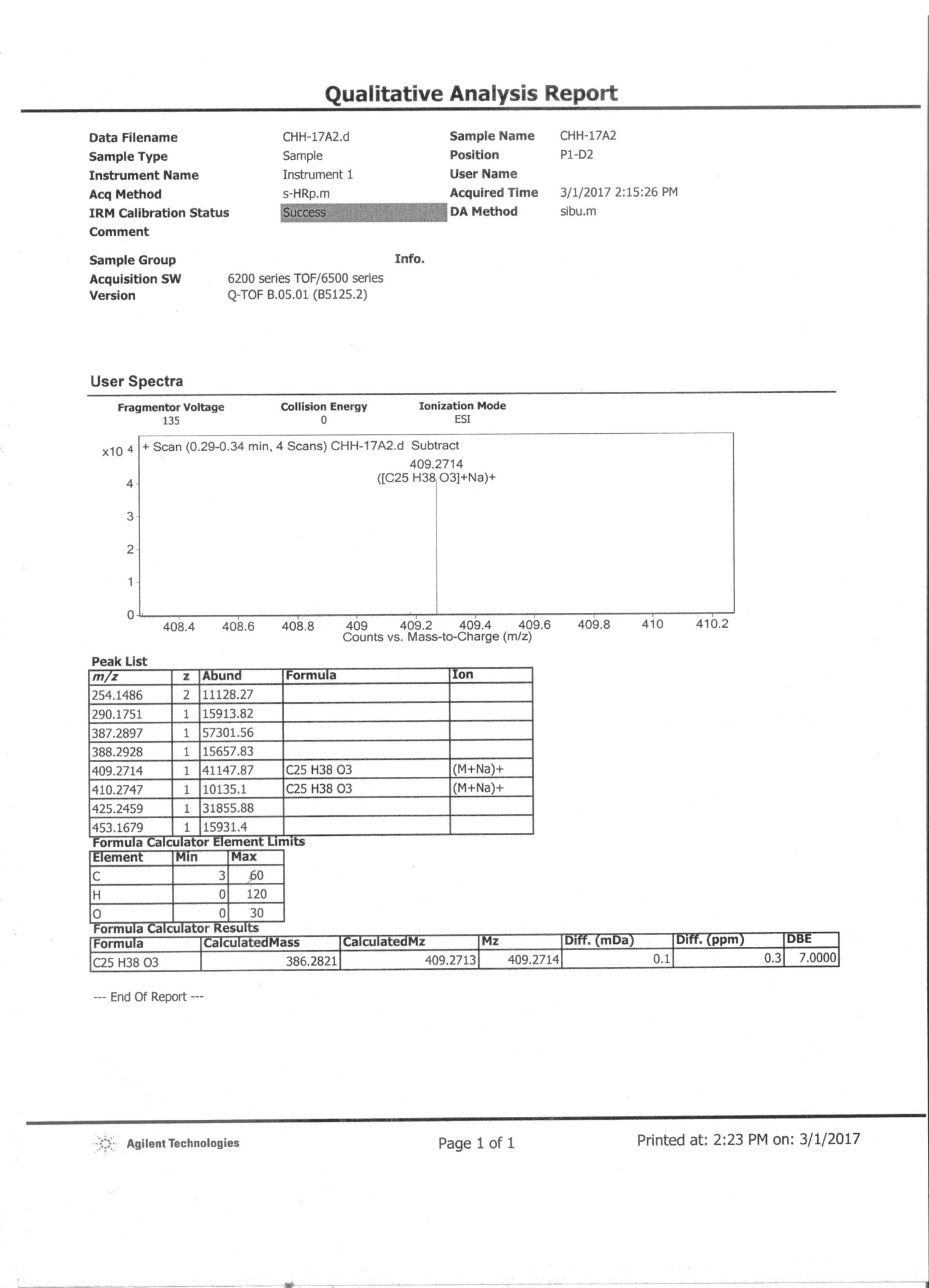


Figure S7. HRESIMS spectrum of compound **1**


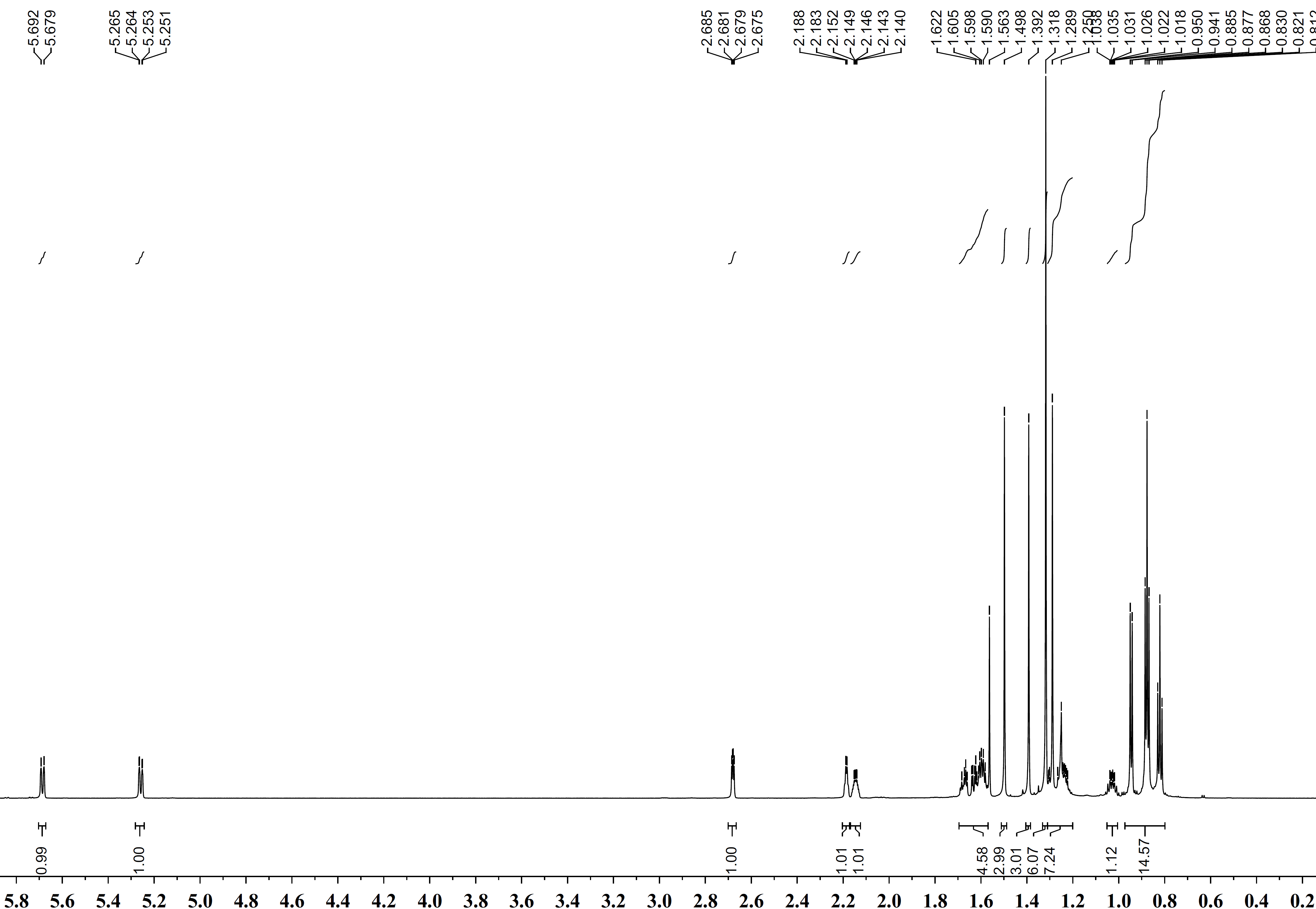


Figure S8. ^1^H NMR spectrum of compound **2** in CHCl_3_


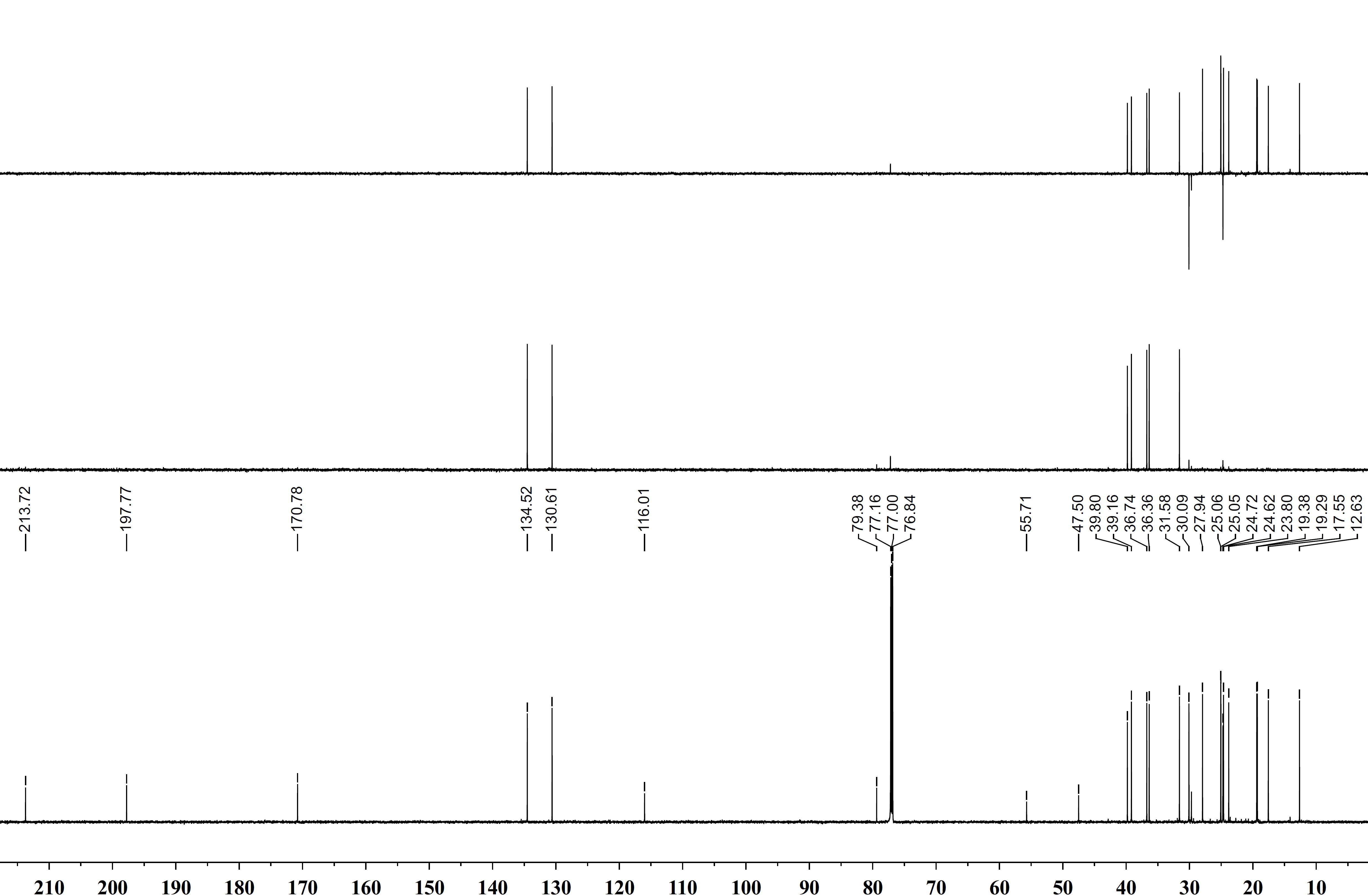


Figure S9. ^13^C NMR spectrum of compound **2** in CHCl_3_


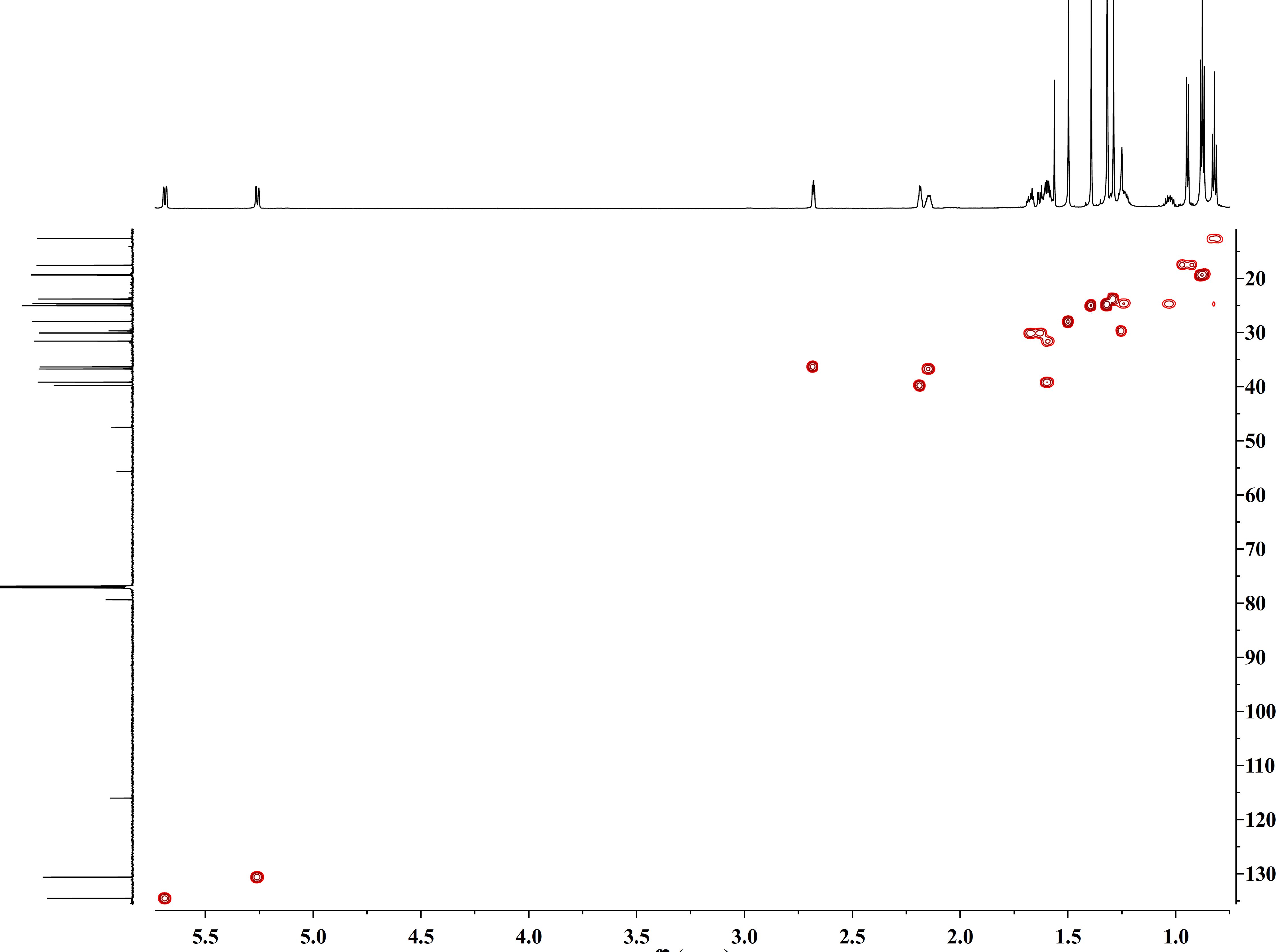


Figure S10. HSQC spectrum of compound **2**


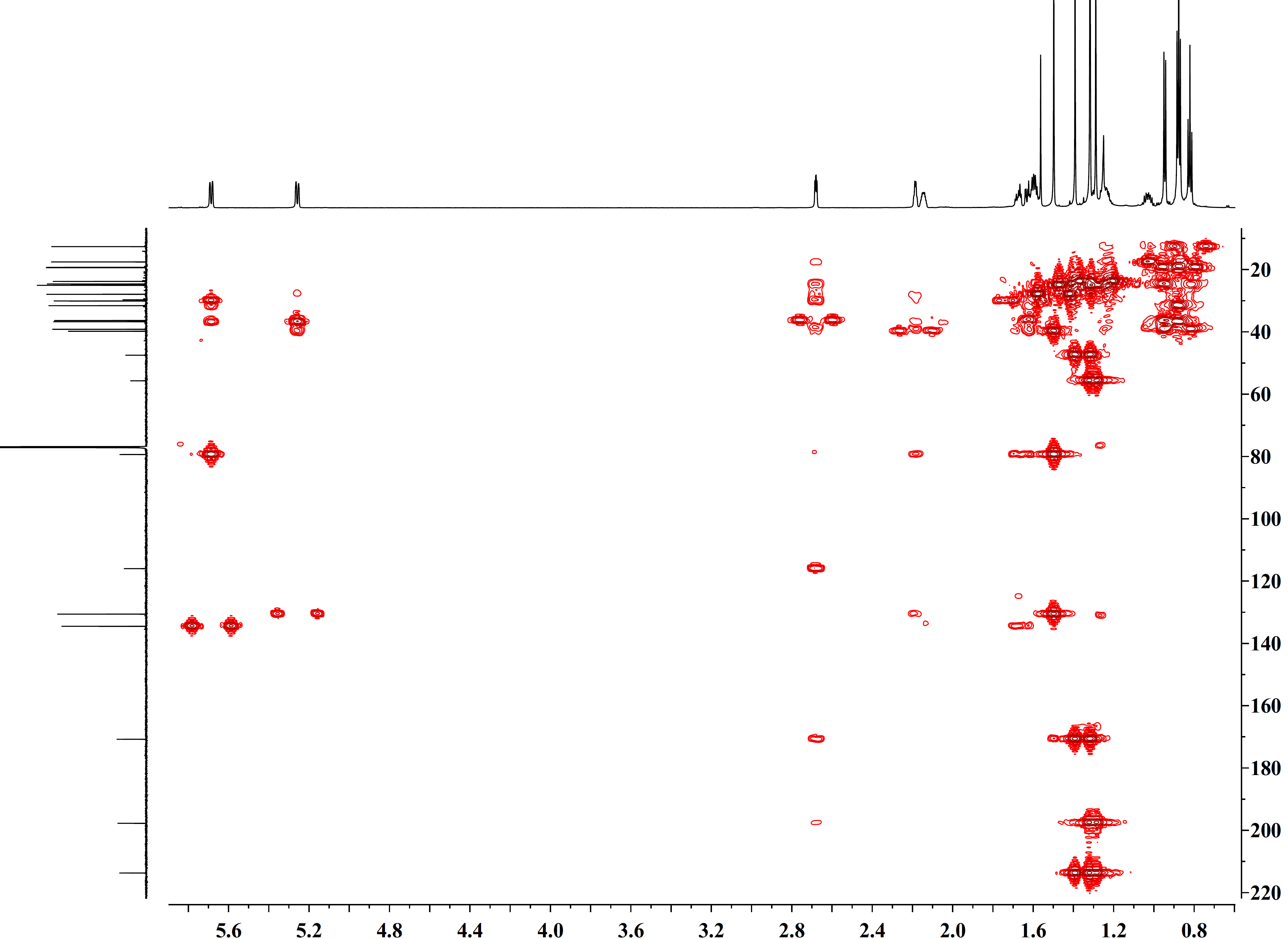


Figure S11. HMBC spectrum of compound **2**


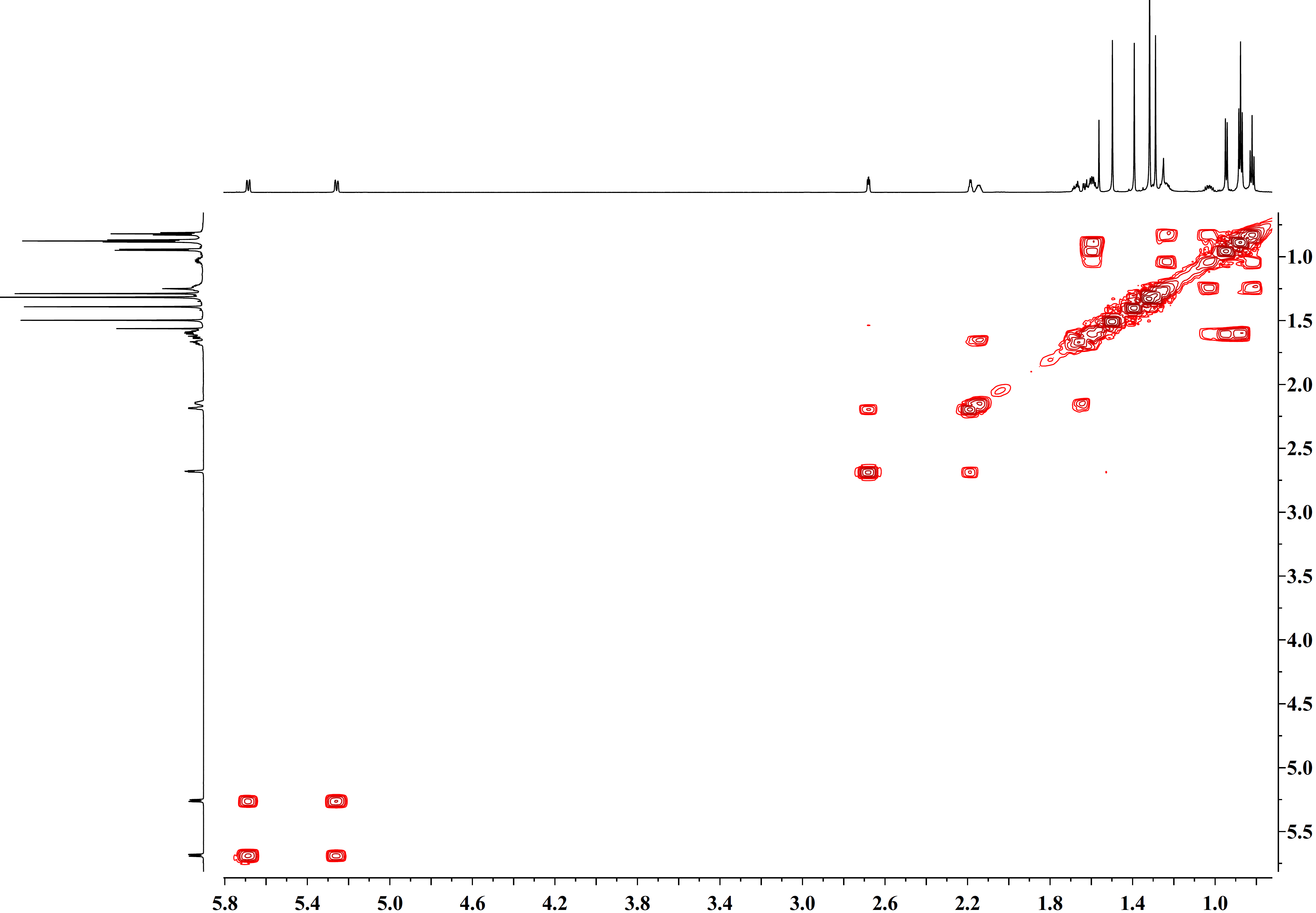


Figure S12. ^1^H‒^1^H COSY spectrum of compound **2**


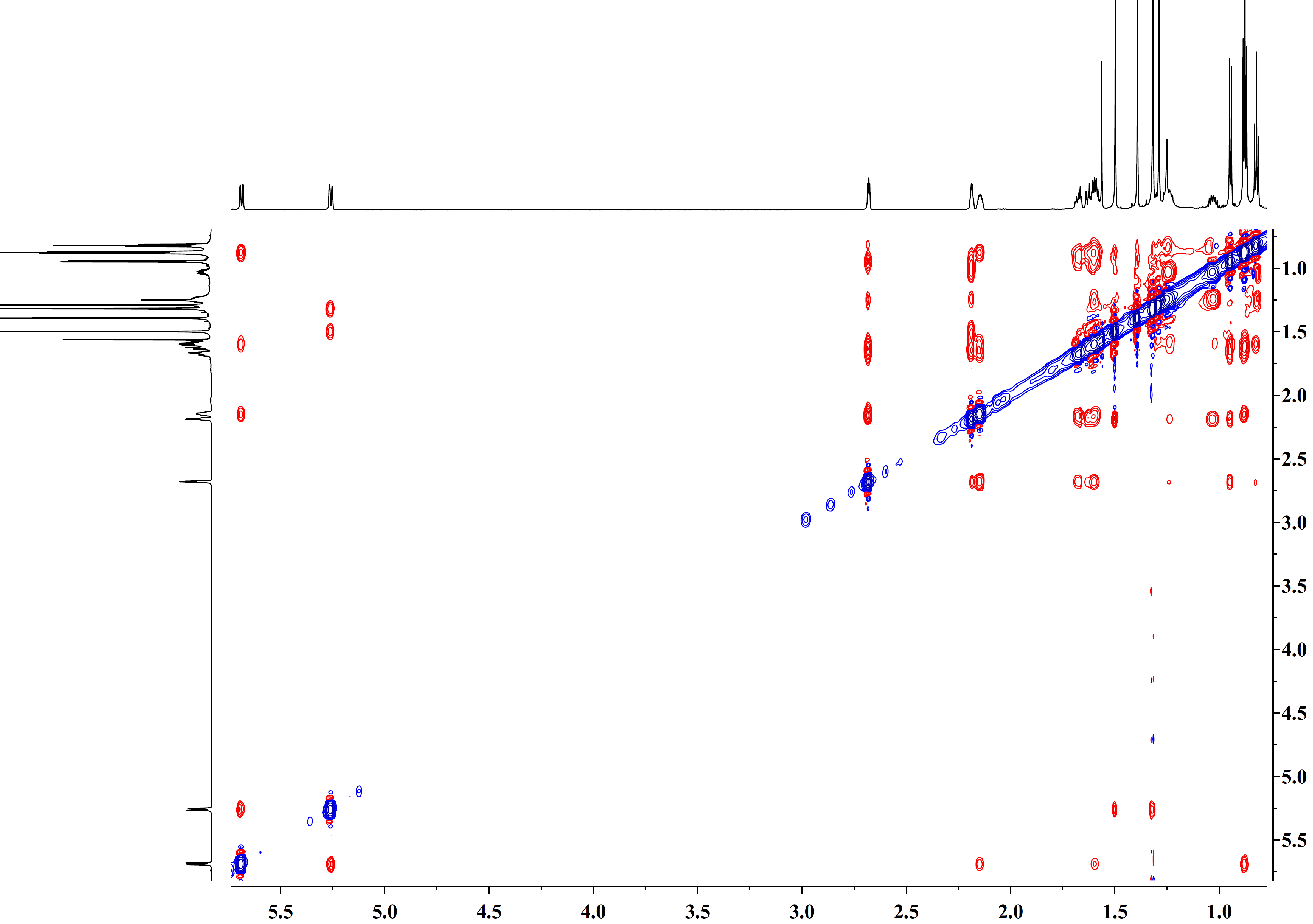


Figure S13. ROESY spectrum of compound **2**


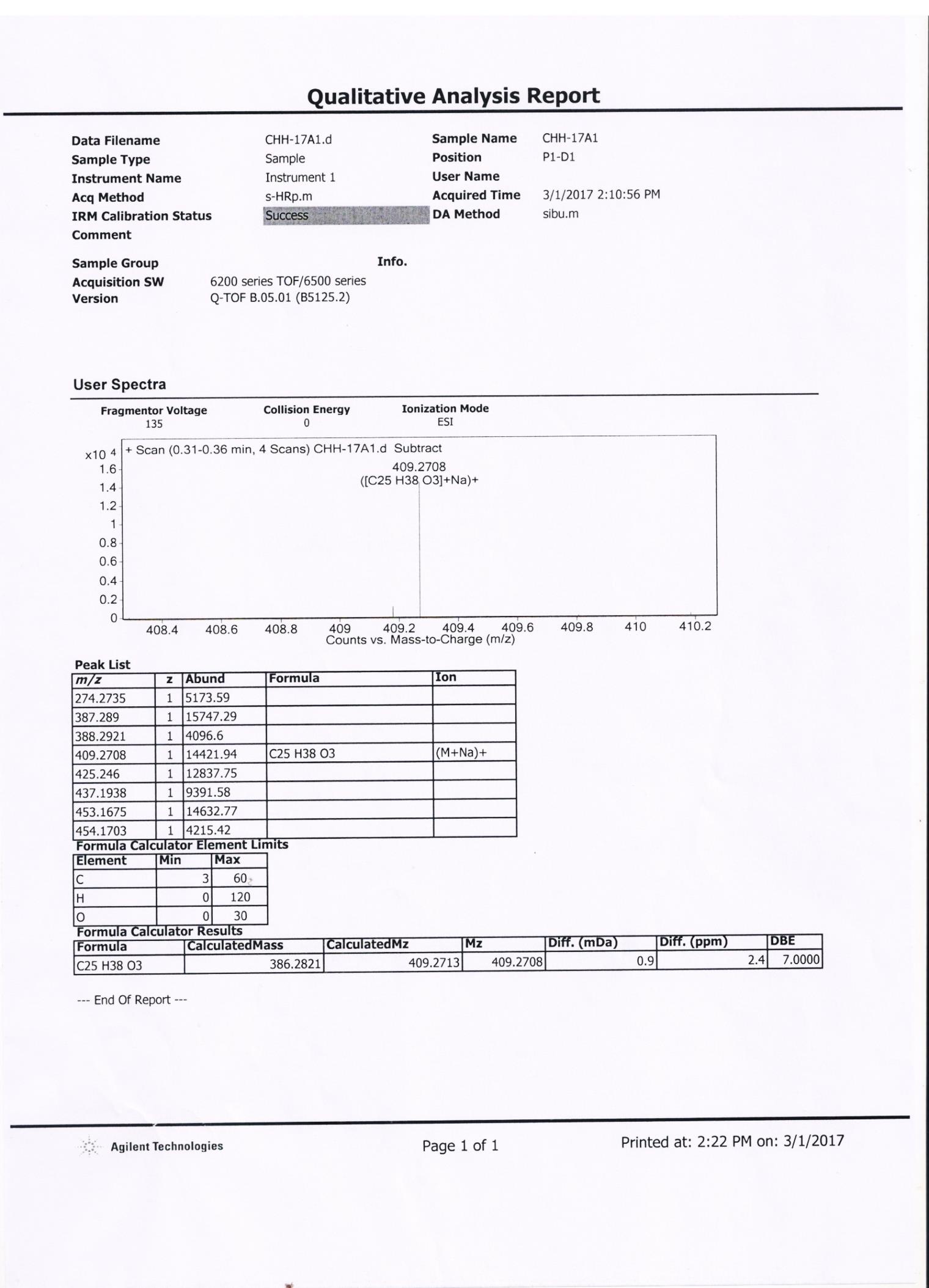


Figure S14. HRESIMS spectrum of compound **2**


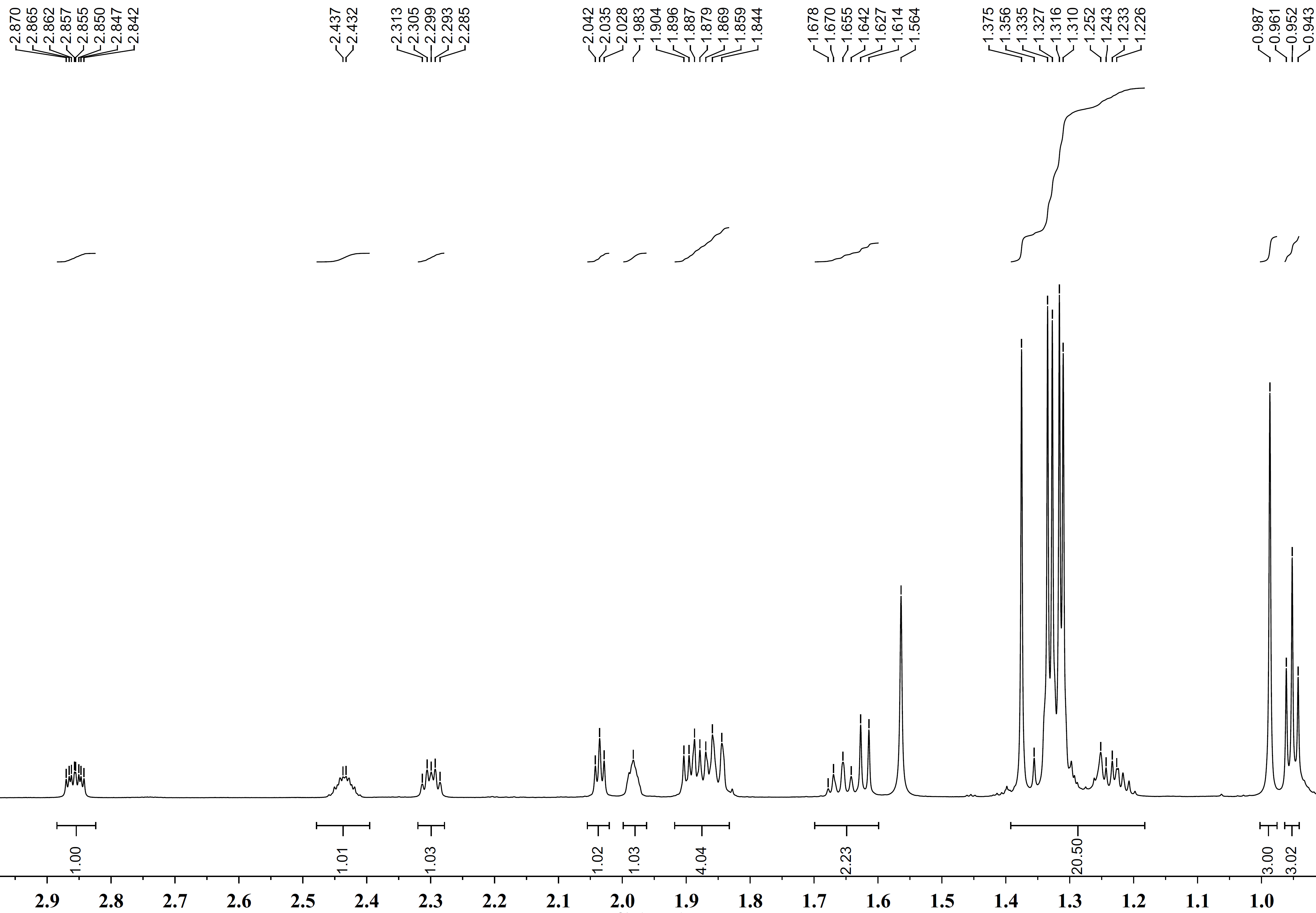


Figure S15. ^1^H NMR spectrum of compound **3** in CDCl_3_


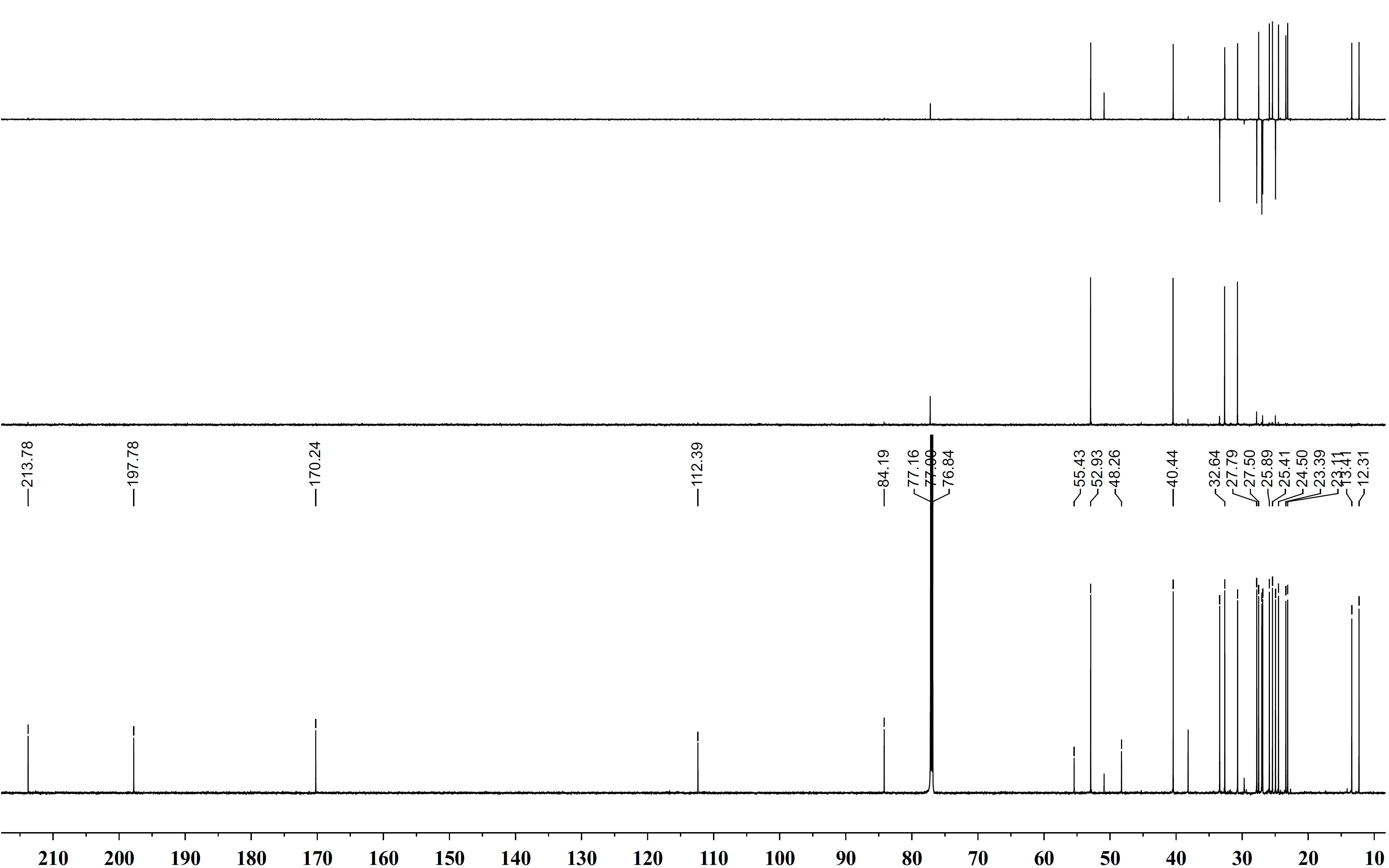


Figure S16. ^13^C NMR spectrum of compound **3** in CDCl_3_


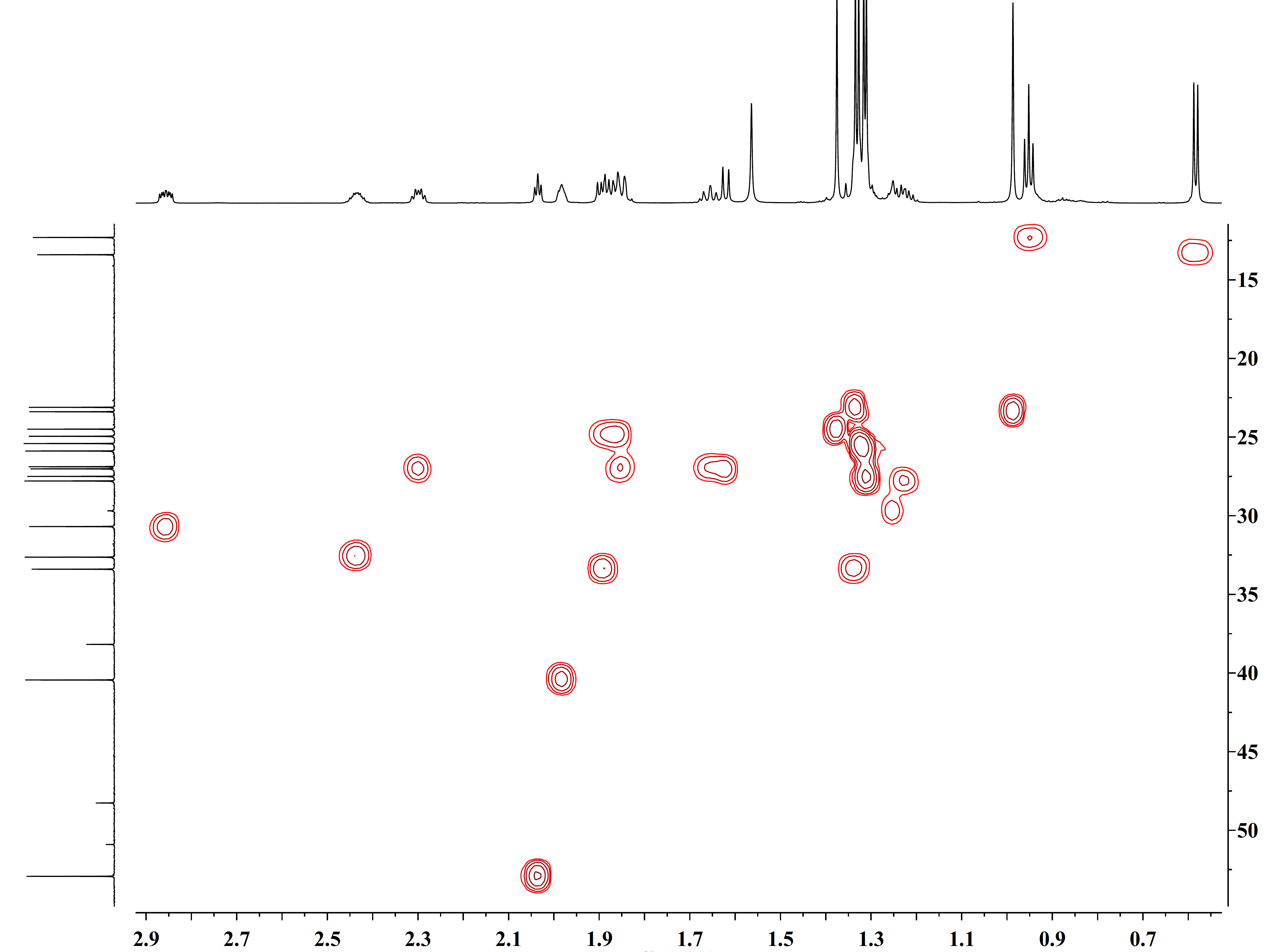


Figure S17. HSQC spectrum of compound **3**


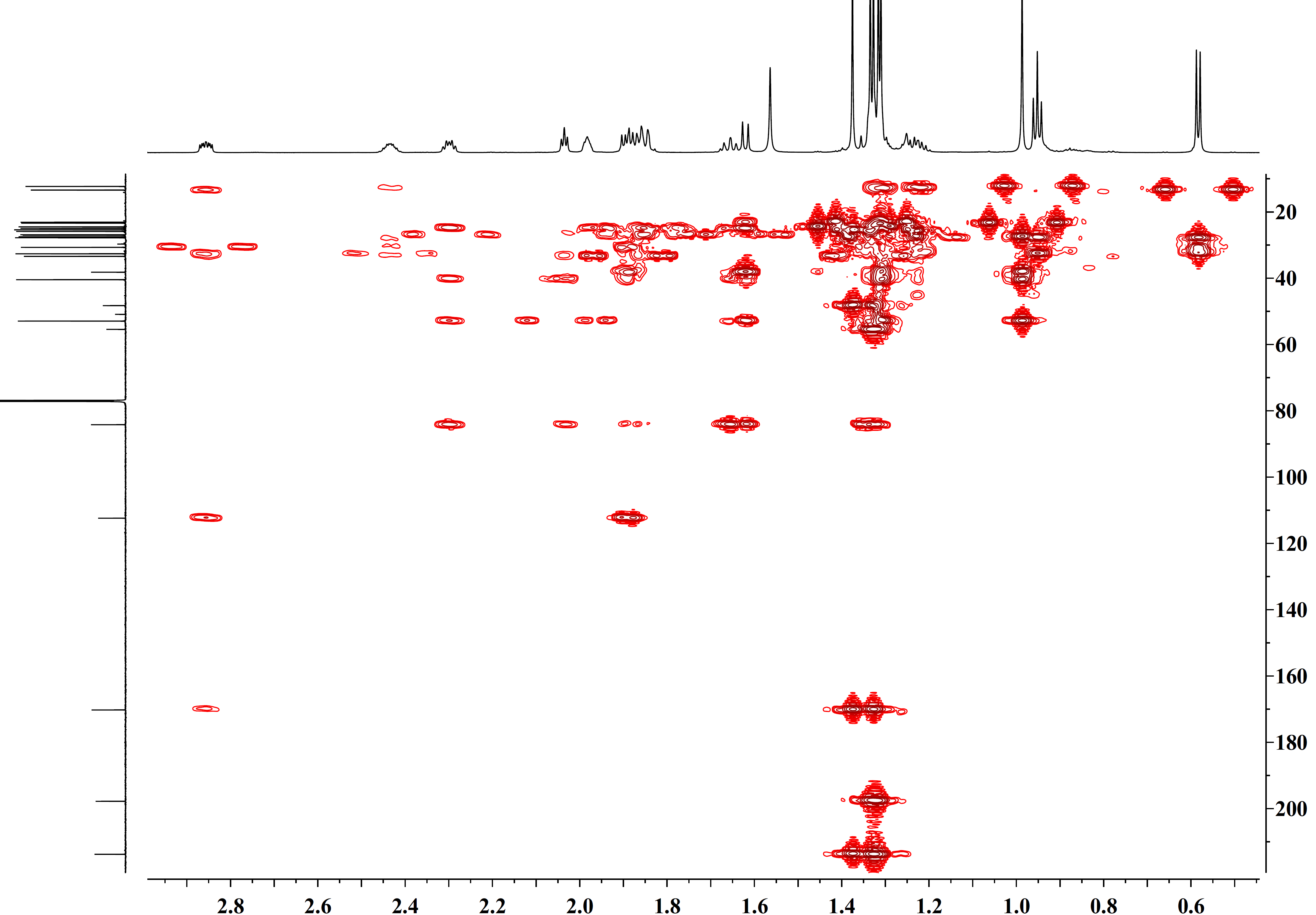


Figure S18. HMBC spectrum of compound **3**


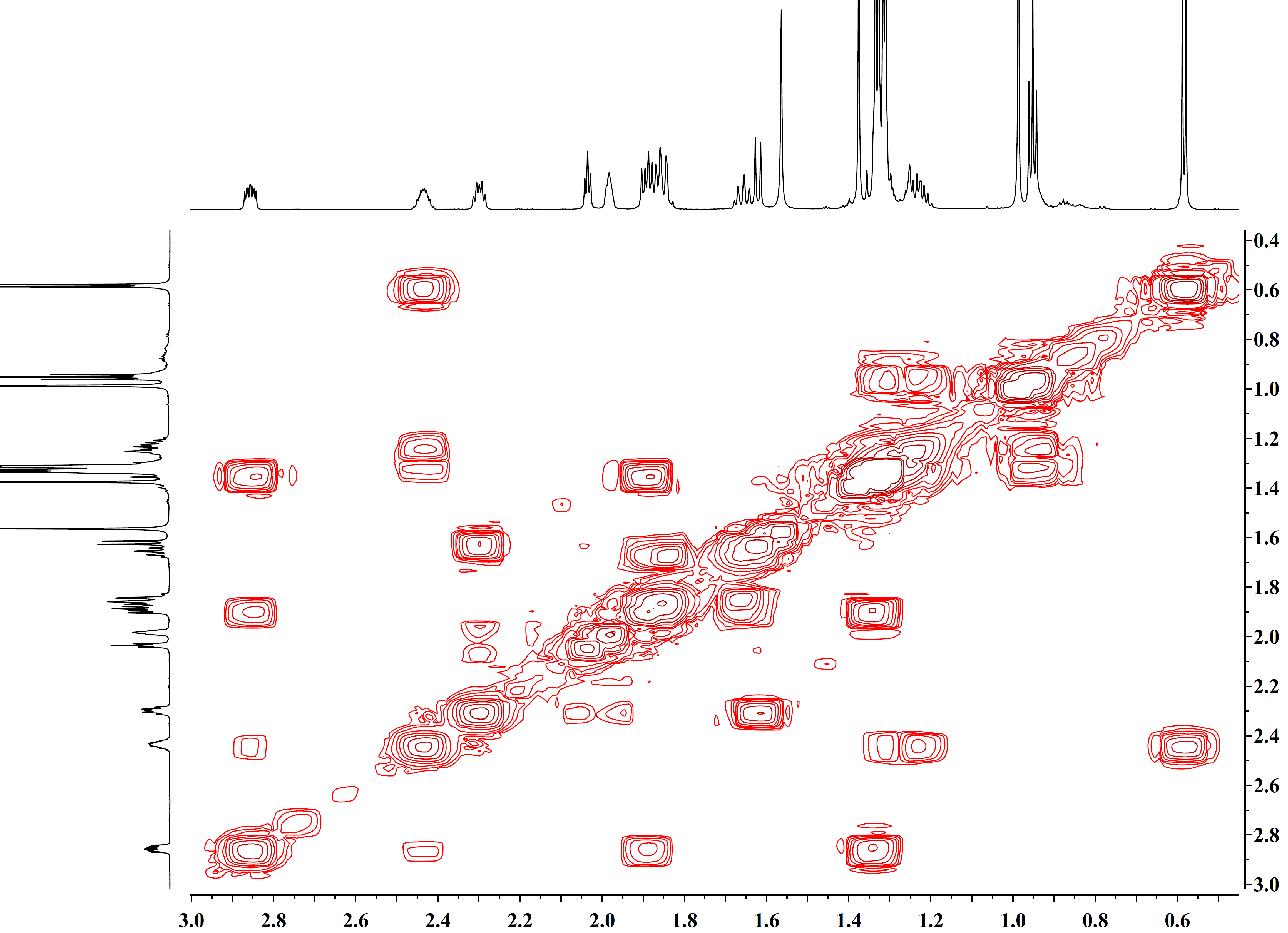


Figure S19. ^1^H‒^1^H COSY spectrum of compound **3**


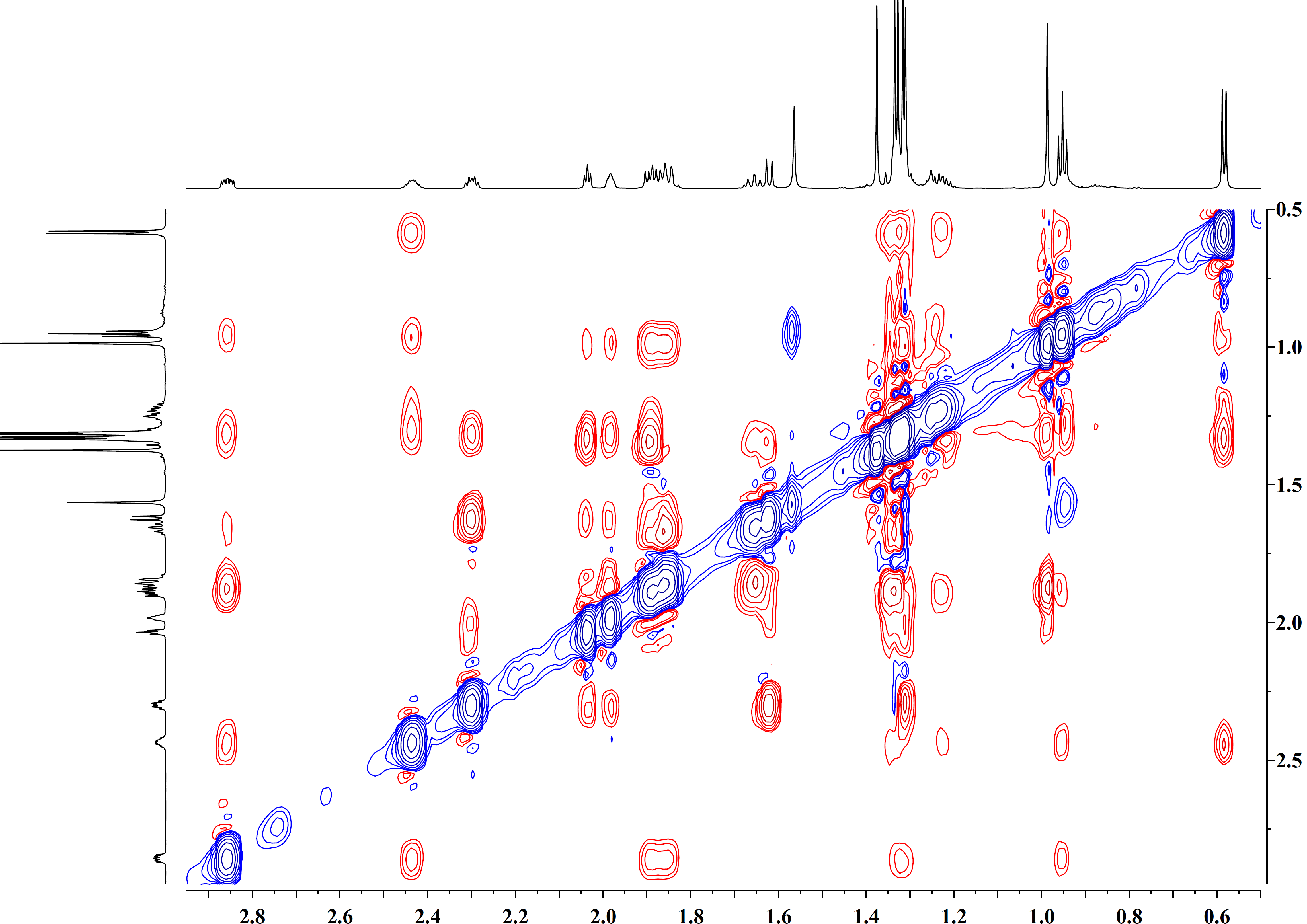


Figure S20. ROESY spectrum of compound **3**


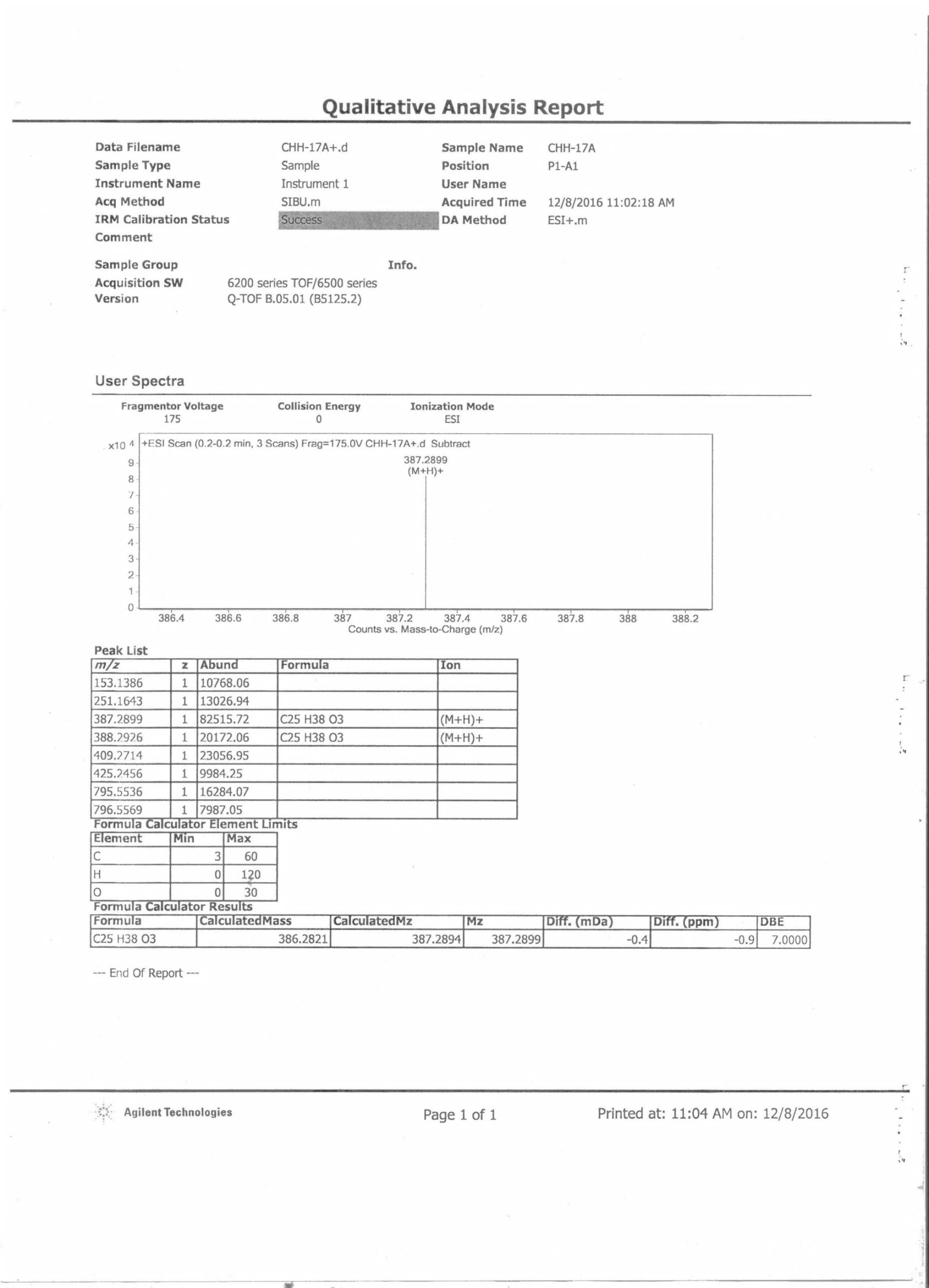


Figure S21. HRESIMS spectrum of compound **3**

Optimized structure of 7*S*,1*'R*,4*'R*,6*'R*–**1**


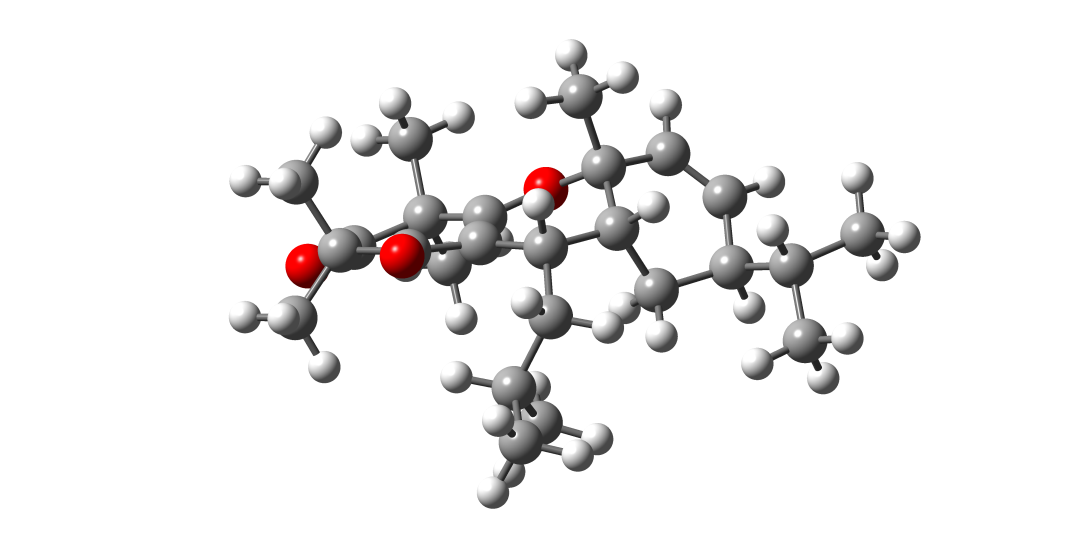


**Standard orientation:**

---------------------------------------------------------------------

Center Atomic Atomic Coordinates (Angstroms)

Number Number Type X Y Z

---------------------------------------------------------------------

1 6 0 -3.432461 -0.776667 0.759735

2 6 0 -3.581449 0.310128 -0.302742

3 6 0 -2.248769 0.817313 -0.877980

4 6 0 -0.982341 0.192213 -0.505610

5 6 0 -0.999878 -1.028305 0.086213

6 6 0 -2.173004 -1.659065 0.804464

7 6 0 0.296377 0.785755 -1.072448

8 6 0 1.546187 -0.079443 -0.734061

9 6 0 1.190679 -1.572654 -0.795577

10 8 0 0.072364 -1.837308 0.133375

11 6 0 2.264884 0.207650 0.592172

12 6 0 3.600678 -0.553311 0.710432

13 6 0 3.379783 -1.998499 0.349788

14 6 0 2.306539 -2.449271 -0.288374

15 6 0 0.761831 -2.039992 -2.188899

16 6 0 0.496304 2.321463 -0.824898

17 8 0 -2.283614 1.723949 -1.699642

18 8 0 -4.332091 -0.996839 1.538188

19 6 0 -2.535731 -3.025472 0.167855

20 6 0 -1.788077 -1.883888 2.285888

21 6 0 -4.407105 -0.284890 -1.473184

22 6 0 -4.345669 1.507545 0.294667

23 6 0 4.783041 0.093658 -0.073262

24 6 0 6.041328 -0.785477 -0.050539

25 6 0 5.126064 1.488218 0.466977

26 1 0 2.263827 0.103710 -1.540152

27 6 0 -0.166591 3.047117 0.367863

28 6 0 0.251290 2.586527 1.767930

29 6 0 0.081084 4.555711 0.221261

30 1 0 0.187865 0.718466 -2.159537

31 1 0 1.625501 -0.095565 1.424193

32 1 0 2.435245 1.273846 0.706518

33 1 0 3.899981 -0.533462 1.767665

34 1 0 4.137202 -2.712225 0.656971

35 1 0 2.191806 -3.512128 -0.478595

36 1 0 1.581855 -1.894010 -2.894661

37 1 0 0.515095 -3.102911 -2.166819

38 1 0 -0.107319 -1.493096 -2.552723

39 1 0 0.117340 2.823546 -1.714792

40 1 0 1.573539 2.521831 -0.804519

41 1 0 -3.395898 -3.448262 0.687470

42 1 0 -1.693413 -3.710217 0.259499

43 1 0 -2.783843 -2.929310 -0.890664

44 1 0 -2.642967 -2.297219 2.817900

45 1 0 -1.510236 -0.943538 2.766428

46 1 0 -0.945184 -2.570088 2.354765

47 1 0 -5.383716 -0.609241 -1.111426

48 1 0 -3.907668 -1.138180 -1.936507

49 1 0 -4.537813 0.484100 -2.234355

50 1 0 -4.481491 2.267581 -0.473160

51 1 0 -5.313306 1.179056 0.670030

52 1 0 -3.794411 1.951818 1.126536

53 1 0 4.474640 0.198864 -1.119972

54 1 0 6.868374 -0.280680 -0.554891

55 1 0 5.886561 -1.742103 -0.551102

56 1 0 6.359436 -0.989252 0.977159

57 1 0 5.959308 1.920381 -0.092107

58 1 0 4.291668 2.186904 0.395738

59 1 0 5.427372 1.433310 1.517845

60 1 0 -1.243937 2.895339 0.279620

61 1 0 -0.311697 3.138637 2.525436

62 1 0 0.060476 1.524961 1.927017

63 1 0 1.312730 2.772215 1.955235

64 1 0 -0.421704 5.114848 1.014397

65 1 0 -0.288186 4.928470 -0.736876

66 1 0 1.149733 4.787018 0.281042

-----------------------------------------------------------------------

Optimized structure of 7*S*,8*R*,1*'S*,4*'S*,6*'S*-**2**


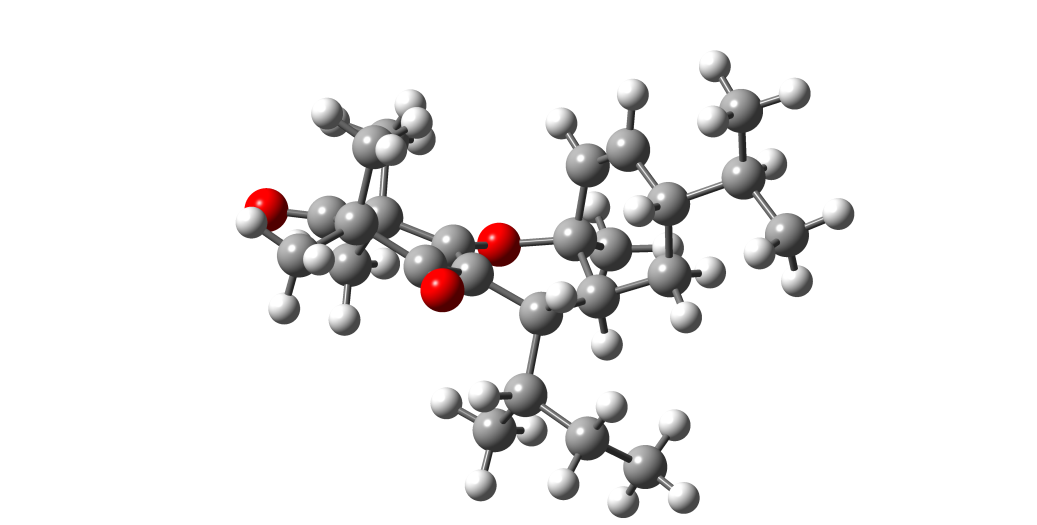


Standard orientation:

---------------------------------------------------------------------

Center Atomic Atomic Coordinates (Angstroms)

Number Number Type X Y Z

---------------------------------------------------------------------

1 6 0 -3.610615 -0.520223 -0.198136

2 6 0 -2.932515 -0.448101 -1.566151

3 6 0 -1.581067 0.288779 -1.536449

4 6 0 -0.834953 0.386275 -0.283551

5 6 0 -1.345460 -0.113501 0.861043

6 6 0 -2.766967 -0.571743 1.089792

7 6 0 0.525941 1.050349 -0.324335

8 6 0 1.446657 0.667136 0.871980

9 6 0 0.844223 -0.381737 1.826242

10 8 0 -0.606441 -0.194268 1.985988

11 6 0 2.832299 0.185561 0.393179

12 6 0 2.808749 -1.158022 -0.355740

13 6 0 1.911179 -2.128985 0.367187

14 6 0 1.057197 -1.788009 1.325591

15 6 0 1.364300 -0.226908 3.255306

16 6 0 0.339406 2.598227 -0.513617

17 8 0 -1.117977 0.711054 -2.586450

18 8 0 -4.815032 -0.591569 -0.111526

19 6 0 -2.803426 -2.028920 1.614855

20 6 0 -3.426934 0.352233 2.141373

21 6 0 -2.632458 -1.903960 -2.023227

22 6 0 -3.872784 0.217167 -2.581226

23 6 0 4.234048 -1.739275 -0.584295

24 6 0 5.187797 -0.707978 -1.202867

25 6 0 4.209494 -2.995793 -1.465866

26 1 0 1.602940 1.563535 1.472813

27 6 0 1.613160 3.343933 -0.989685

28 6 0 -0.336422 3.297059 0.670991

29 6 0 2.623978 3.819687 0.061575

30 1 0 0.994951 0.714722 -1.251982

31 1 0 3.500167 0.089766 1.256257

32 1 0 3.263582 0.957118 -0.244285

33 1 0 2.382471 -0.994992 -1.356821

34 1 0 1.970246 -3.173966 0.083016

35 1 0 0.456570 -2.547506 1.817002

36 1 0 2.449822 -0.319409 3.282861

37 1 0 0.939356 -0.998025 3.899918

38 1 0 1.079988 0.749548 3.652062

39 1 0 -0.347351 2.669610 -1.358857

40 1 0 -3.841173 -2.336411 1.745121

41 1 0 -2.292184 -2.088347 2.575134

42 1 0 -2.320987 -2.723627 0.925543

43 1 0 -4.459298 0.042276 2.293647

44 1 0 -3.428218 1.391585 1.807131

45 1 0 -2.883901 0.293262 3.083985

46 1 0 -3.557161 -2.482452 -2.058747

47 1 0 -1.927264 -2.407448 -1.360223

48 1 0 -2.195122 -1.873258 -3.022060

49 1 0 -3.380849 0.268748 -3.550460

50 1 0 -4.798804 -0.349501 -2.661692

51 1 0 -4.126982 1.233129 -2.272895

52 1 0 4.632075 -2.020391 0.399328

53 1 0 6.160593 -1.163069 -1.402655

54 1 0 5.356703 0.149844 -0.551271

55 1 0 4.797195 -0.335609 -2.155238

56 1 0 3.801086 -2.767332 -2.455074

57 1 0 5.221910 -3.380374 -1.608786

58 1 0 3.615308 -3.804216 -1.038209

59 1 0 2.122540 2.722103 -1.733122

60 1 0 1.273791 4.229935 -1.536786

61 1 0 -1.304242 2.845783 0.894536

62 1 0 0.261691 3.265140 1.584275

63 1 0 -0.512574 4.350539 0.437820

64 1 0 3.069915 3.002936 0.630388

65 1 0 2.168211 4.508282 0.776039

66 1 0 3.441635 4.356814 -0.425609

-----------------------------------------------------------------------

Optimized structure of 7*R*,8*S*,1*'S*,2*'S*,4*'R*-**3**


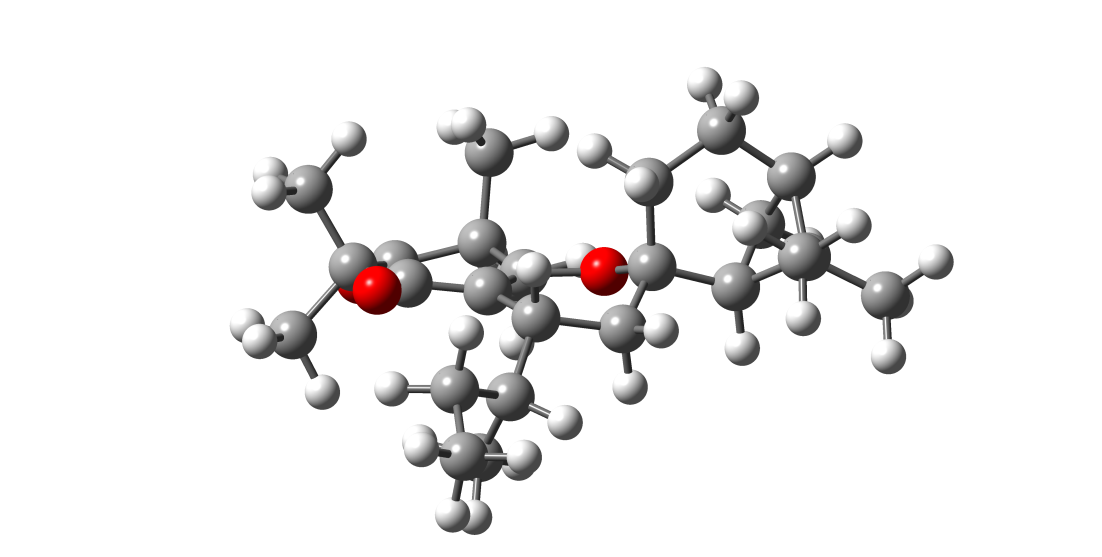


Standard orientation:

---------------------------------------------------------------------

Center Atomic Atomic Coordinates (Angstroms)

Number Number Type X Y Z

---------------------------------------------------------------------

1 6 0 2.750919 -2.047420 -0.420918

2 6 0 3.368838 -0.888277 0.345286

3 6 0 2.407347 0.279673 0.558040

4 6 0 1.105981 0.264887 0.164071

5 6 0 0.527903 -0.871691 -0.275789

6 6 0 1.242376 -2.214546 -0.356467

7 6 0 0.895843 -3.040631 0.898181

8 6 0 0.782216 -2.999739 -1.606189

9 6 0 3.906521 -1.387000 1.698679

10 6 0 4.556429 -0.354926 -0.490864

11 6 0 0.283968 1.547524 0.255783

12 6 0 -1.115905 1.311565 -0.354120

13 6 0 -1.694116 -0.063668 -0.042038

14 8 0 -0.805238 -1.004299 -0.554303

15 6 0 -3.010472 -0.281797 -0.811367

16 6 0 -4.331151 0.206753 -0.141560

17 6 0 -4.231359 -1.099748 0.691435

18 6 0 -3.218657 -1.031052 1.842979

19 6 0 -1.896116 -0.324347 1.467052

20 8 0 3.443988 -2.801829 -1.067909

21 8 0 2.872952 1.258882 1.094825

22 6 0 0.868200 2.807920 -0.460953

23 6 0 1.609834 2.461236 -1.765491

24 6 0 1.696567 3.767810 0.423535

25 6 0 1.643101 5.230768 -0.047023

26 6 0 -5.525309 0.235015 -1.119684

27 6 0 -4.361059 1.520942 0.646497

28 6 0 -3.514656 -1.730186 -0.528239

29 1 0 0.144563 1.767849 1.341870

30 1 0 1.472637 -3.992503 0.931982

31 1 0 -0.185218 -3.306960 0.926505

32 1 0 1.106317 -2.479947 1.835136

33 1 0 1.282606 -3.991578 -1.684087

34 1 0 -0.309531 -3.216137 -1.593464

35 1 0 0.998897 -2.429465 -2.538387

36 1 0 4.561005 -2.278746 1.569859

37 1 0 3.086849 -1.667189 2.395130

38 1 0 4.512969 -0.606398 2.211251

39 1 0 5.344531 -1.127314 -0.639176

40 1 0 4.217425 -0.018293 -1.497622

41 1 0 5.059988 0.510698 -0.004525

42 1 0 -1.812896 2.114668 -0.024993

43 1 0 -1.054831 1.391848 -1.465548

44 1 0 -2.940689 -0.052334 -1.896954

45 1 0 -5.203273 -1.568758 0.954159

46 1 0 -3.694486 -0.530823 2.718791

47 1 0 -3.003210 -2.077303 2.167724

48 1 0 -1.841337 0.641244 2.018589

49 1 0 -1.040618 -0.896255 1.895502

50 1 0 -0.015049 3.435264 -0.750871

51 1 0 1.809272 3.368943 -2.377554

52 1 0 1.022990 1.764153 -2.403673

53 1 0 2.593593 1.982847 -1.567277

54 1 0 2.768567 3.467355 0.437730

55 1 0 1.324094 3.737106 1.475049

56 1 0 2.278437 5.880024 0.599131

57 1 0 0.605688 5.634302 -0.003060

58 1 0 2.011922 5.341900 -1.091427

59 1 0 -6.485883 0.312885 -0.561854

60 1 0 -5.450377 1.117199 -1.795195

61 1 0 -5.615092 -0.652583 -1.779450

62 1 0 -3.588507 1.624079 1.430067

63 1 0 -4.250584 2.391130 -0.038851

64 1 0 -5.335682 1.627057 1.174358

65 1 0 -4.199178 -2.138841 -1.301528

66 1 0 -2.752968 -2.495554 -0.273257

-----------------------------------------------------------------------

Optimized structure of 8*S*,7*'S*-**4**


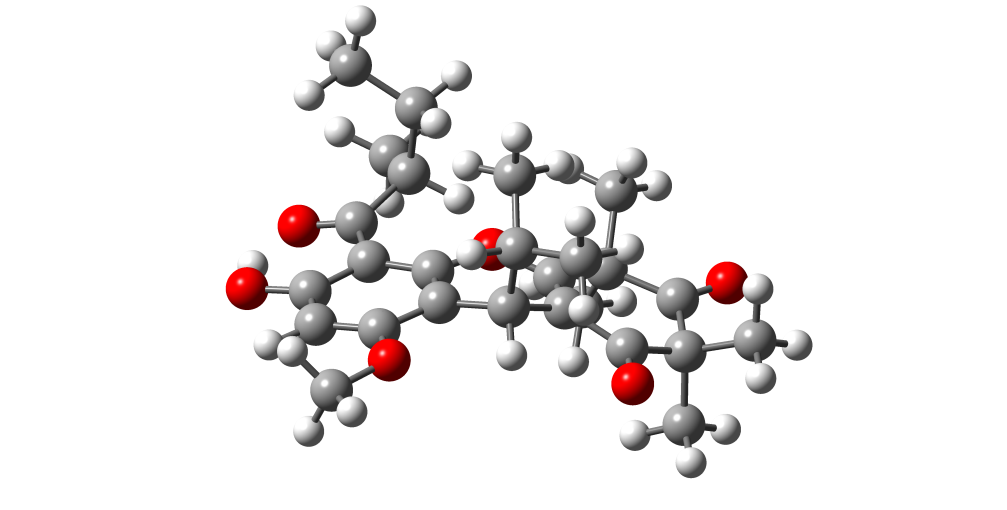


Standard orientation:

---------------------------------------------------------------------

Center Atomic Atomic Coordinates (Angstroms)

Number Number Type X Y Z

---------------------------------------------------------------------

1 6 0 3.206506 1.206657 -0.993970

2 6 0 2.664517 2.493065 -0.942599

3 6 0 1.366235 2.677775 -0.510571

4 6 0 0.557696 1.581503 -0.133730

5 6 0 1.106055 0.321048 -0.213708

6 6 0 2.453956 0.059997 -0.596531

7 6 0 3.151153 -1.239458 -0.581015

8 8 0 4.275228 -1.332289 -1.098332

9 6 0 2.617411 -2.474700 0.135519

10 6 0 3.345101 -3.740681 -0.324574

11 6 0 2.694399 -2.268923 1.673541

12 6 0 4.100341 -2.051623 2.237359

13 8 0 4.463931 1.116216 -1.421732

14 8 0 0.768366 3.887644 -0.419210

15 6 0 1.498723 5.045766 -0.812655

16 6 0 -0.853248 1.787377 0.366185

17 6 0 -1.663443 0.552961 0.047288

18 6 0 -1.063499 -0.646801 -0.044434

19 8 0 0.295933 -0.775959 0.048245

20 6 0 -0.866928 2.206078 1.878343

21 6 0 -0.282340 1.147857 2.818853

22 6 0 -2.255379 2.643973 2.356186

23 6 0 -3.080990 0.707043 -0.298366

24 6 0 -3.937658 -0.523898 -0.627201

25 6 0 -3.265896 -1.860784 -0.315588

26 6 0 -1.727865 -1.986029 -0.241344

27 6 0 -1.262486 -2.642195 -1.567603

28 6 0 -1.385782 -2.920113 0.942642

29 6 0 -4.220729 -0.488733 -2.153819

30 6 0 -5.270492 -0.428347 0.138361

31 8 0 -3.934592 -2.859427 -0.185178

32 8 0 -3.587438 1.813024 -0.384971

33 1 0 3.301246 3.309415 -1.247097

34 1 0 1.562921 -2.574424 -0.108187

35 1 0 4.415663 -3.690019 -0.134852

36 1 0 3.215650 -3.898179 -1.396775

37 1 0 2.937967 -4.609467 0.198319

38 1 0 2.249647 -3.153852 2.139780

39 1 0 2.056899 -1.426913 1.951703

40 1 0 4.054624 -1.912415 3.319652

41 1 0 4.577710 -1.167090 1.810175

42 1 0 4.753196 -2.904085 2.042825

43 1 0 4.673789 0.138526 -1.435847

44 1 0 2.388803 5.179304 -0.192788

45 1 0 1.788856 4.989032 -1.864689

46 1 0 0.821870 5.883714 -0.665392

47 1 0 -1.302215 2.618040 -0.178687

48 1 0 -0.215579 3.085182 1.921455

49 1 0 -0.251138 1.531258 3.841480

50 1 0 -0.894611 0.242025 2.830970

51 1 0 0.736229 0.869137 2.542582

52 1 0 -2.186338 3.074528 3.358596

53 1 0 -2.947538 1.800394 2.410333

54 1 0 -2.697109 3.385607 1.690375

55 1 0 -0.191513 -2.840512 -1.539608

56 1 0 -1.789845 -3.586508 -1.703884

57 1 0 -1.466005 -2.003268 -2.428363

58 1 0 -1.931951 -3.854023 0.825224

59 1 0 -1.676743 -2.470727 1.894081

60 1 0 -0.317901 -3.129035 0.974242

61 1 0 -4.855410 -1.331111 -2.431429

62 1 0 -4.731151 0.444282 -2.392503

63 1 0 -3.302917 -0.533559 -2.743304

64 1 0 -5.915231 -1.262191 -0.133223

65 1 0 -5.757381 0.514820 -0.103057

66 1 0 -5.108069 -0.464449 1.217897

---------------------------------------------------------------------
